# Bcl-2 dependent modulation of Hippo pathway in cancer cells

**DOI:** 10.1186/s12964-024-01647-1

**Published:** 2024-05-16

**Authors:** Simona D’Aguanno, Matteo Brignone, Stefano Scalera, Martina Chiacchiarini, Marta Di Martile, Elisabetta Valentini, Francesca De Nicola, Alessia Ricci, Fabio Pelle, Claudio Botti, Marcello Maugeri-Saccà, Donatella Del Bufalo

**Affiliations:** 1 Preclinical Models and New Therapeutic Agents Unit, IRCCS Regina Elena National Cancer Institute, Rome, 00144 Italy; 2 Clinical Trial Center, Biostatistics and Bioinformatics, IRCCS Regina Elena National Cancer Institute, Rome, 00144 Italy; 3 SAFU, IRCCS Regina Elena National Cancer Institute, Rome, 00144 Italy; 4 Department of Pharmacy, University “G. d’Annunzio” of Chieti-Pescara, Chieti, 66100 Italy; 5 Department of Surgery, Division of Breast Surgery, IRCCS Regina Elena National Cancer Institute, Rome, 00144 Italy

**Keywords:** Bcl-2, Melanoma, Breast cancer, Hippo Pathway

## Abstract

**Introduction:**

Bcl-2 and Bcl-xL are the most studied anti-apoptotic members of Bcl-2 family proteins. We previously characterized both of them, not only for their role in regulating apoptosis and resistance to therapy in cancer cells, but also for their non-canonical functions, mainly including promotion of cancer progression, metastatization, angiogenesis, and involvement in the crosstalk among cancer cells and components of the tumor microenvironment. Our goal was to identify transcriptional signature and novel cellular pathways specifically modulated by Bcl-2.

**Methods:**

We performed RNAseq analysis of siRNA-mediated transient knockdown of Bcl-2 or Bcl-xL in human melanoma cells and gene ontology analysis to identify a specific Bcl-2 transcriptional signature. Expression of genes modulated by Bcl-2 and associated to Hippo pathway were validated in human melanoma, breast adenocarcinoma and non-small cell lung cancer cell lines by qRT-PCR. Western blotting analysis were performed to analyse protein expression of upstream regulators of YAP and in relation to different level of Bcl-2 protein. The effects of YAP silencing in Bcl-2 overexpressing cancer cells were evaluated in migration and cell viability assays in relation to different stiffness conditions. In vitro wound healing assays and co-cultures were used to evaluate cancer-specific Bcl-2 ability to activate fibroblasts.

**Results:**

We demonstrated the Bcl-2-dependent modulation of Hippo Pathway in cancer cell lines from different tumor types by acting on upstream YAP regulators. YAP inhibition abolished the ability of Bcl-2 to increase tumor cell migration and proliferation on high stiffness condition of culture, to stimulate in vitro fibroblasts migration and to induce fibroblasts activation.

**Conclusions:**

We discovered that Bcl-2 regulates the Hippo pathway in different tumor types, promoting cell migration, adaptation to higher stiffness culture condition and fibroblast activation. Our data indicate that Bcl-2 inhibitors should be further investigated to counteract cancer-promoting mechanisms.

**Supplementary Information:**

The online version contains supplementary material available at 10.1186/s12964-024-01647-1.

## Introduction

Disregulation of apoptosis-mediated cell death can cause cancer and affect the response to treatments [1]. Bcl-2 family proteins are relevant regulators of apoptotic cell death *via* the intrinsic pathway [2–4]. The balance between anti- and pro-apoptotic members belonging to this family is responsible for the cell fate [5]. The most studied anti-apoptotic Bcl-2 family proteins are Bcl-2, Bcl-xL and Mcl-1. Bcl-xL has 44% sequence homology with Bcl-2, while Mcl-1 is a unique member of the Bcl-2 family, with a large size and a short half-life due to the presence of a PEST sequence at its N-terminus [6]. In addition to high sequence homology and similar role in regulating apoptosis and response to therapy, Bcl-2 and Bcl-xL also have common non-canonical roles that elicit tumour-promoting effect [7–9]. In particular, using in vitro and in vivo preclinical cutaneous melanoma (hereafter melanoma) and breast carcinoma models we previously demonstrated Bcl-2 modulation of tumor progression-associated properties and tumor metastatization [10–12]. In melanoma cells, we also described the Bcl-2-mediated regulation of microRNA-211 and mitochondrial transcript levels through the interaction with SLIRP [13, 14]. Likewise, we described the pivotal role played by Bcl-2 in orchestrating the crosstalk between melanoma cells and tumor microenvironment (TME) components, such as neovascular endothelial cells, through a mechanism involving vascular endothelial growth factor (VEGF), and tumor-associated macrophages through interleukin-1β induction of the M2 phenotype [11, 15, 16]. Regarding Bcl-xL, we reported its role in the modulation of properties strictly related to melanoma progression and maintenance of cancer stem cell phenotype, [17]. Bcl-xL also regulates in vitro endothelial cell functions and in vivo vessel formation in cancer models, with a mechanism involving the nuclear factor κB (NF-κB)/IL-8 axis [18]. Moreover, it sustains and induces melanoma aggressiveness, via an autocrine pathway involving IL-8 and its receptor C-X-C motif chemokine receptor 2 (CXCR2) and recruits macrophages at the tumor site by inducing a M2 phenotype in in vivo zebrafish and mouse models of melanoma [19]. In order to identify a Bcl-2 dependent transcriptome signature able to better discriminate between Bcl-2 and Bcl-xL functions, we performed RNAseq analysis of siRNA-mediated transient knockdown of Bcl-2 or Bcl-xL in human melanoma cells. Among the peculiar Bcl-2 modulated pathways, we focused on Hippo Pathway, a highly conserved signalling pathway among organisms. Studies of Hippo pathway in human tumors clarified that its deregulation leads to YAP/TAZ activation, which in turn promotes the development and progression of different types of cancer [20]. This pathway consists of a kinase cascade that begins with MST1/2 which phosphorylates LATS1/2, large protein kinases with tumor suppressive activity [21]. MST1/2 can be phosphorylated by TAO1/2/3 or the modification can occur by trans-phosphorylation of MST1/2 activated loops [20]. After phosphorylation, MST1/2 in turn activates SAV1 and MOB1, scaffold proteins that help MST1/2 in the recruitment and in the phosphorylation of LATS1/2 [22]. Activation of LATS1/2 causes phosphorylation of human YAP and TAZ proteins contributing to maintain YAP/TAZ in the cytoplasm, making them transcriptionally inactive, while the non-phosphorylated forms translocate into the nucleus. Through the cooperation with transcription factors belonging to the TEA domain family (TEAD1- 4) or to other families, such as TP73, YAP/TAZ activate or repress the expression of target genes, mainly involved in tumor progression, drug resistance, and immune response [21]. While the deregulation of Hippo pathway has been reported in many solid tumors, such as lung and breast carcinoma [23], the role of Hippo/YAP signalling in melanoma is not completely elucidated. Several studies reported that YAP/TAZ expression is elevated in benign and dysplastic nevi and in situ melanoma, without significant differences between lesion types [24, 25] and substantial variations in the proportion between the cytoplasmic fraction and the nuclear fraction of YAP/TAZ. The transcriptional signature of YAP in melanoma cells has been recently generated, demonstrating that YAP activation: (i) is elevated in melanoma cells with a more invasive phenotype [23]; (ii) promotes spontaneous metastasis in murine xenograft melanoma model [23]; (iii) can switch melanoma cells from proliferative to invasive phenotypes [23].

Here, we demonstrated the Bcl-2-dependent modulation of genes associated to Hippo Pathway and regulation of core proteins upstream to YAP in cancer cell lines from different histotypes, and the functional significance of this novel cellular signal regulation mediated by Bcl-2.

## Methods

### Cell culture, co-culture, transfection and treatment

Human melanoma A375 and M14, non-small cell lung cancer (NSCLC) H460, and breast adenocarcinoma MDA-MB-231 cell lines were maintained in RPMI-1640 complete medium (Euroclone, Milan, IT) containing 10% inactivated fetal bovine serum (FBS) (GIBCO, ThermoFisher Scientific, Waltham, MA, USA), 1% L-glutamine (Euroclone) and 100 µg/ml penicillin/streptomycin (Euroclone). Human foreskin fibroblasts (HFF), purchased by ATCC and green fluorescent protein (GFP)-labeled HFF, kindly provided by Dr Fabiana Conciatori [26] were maintained in DMEM complete medium (Euroclone) supplemented with 10% FBS, 1% L-glutamine and 100 µg/ml penicillin/streptomycin. Cell lines were routinely tested for mycoplasma contamination and authenticated. Human control and Bcl-2 overexpressing stable melanoma cells were obtained from parental cells (A375 or M14) as previously reported [27] and cultured in the presence of 1 µg/ml puromicine (Sigma-Aldrich, St.Louis, Missouri, USA).

For transient transfection, cells were seeded and, after 24 h, transfected with 20 nM pooled siRNA oligonucleotides against Bcl-2 (si-Bcl-2), or Bcl-xL (si-Bcl-xL), or YAP (si-YAP), or non-targeting control (si-Ctrl) sequences (Horizon Dharmacon ON-TARGETplus siRNA, SMARTpool, Lafayette, Colorado, USA) or with empty or Bcl-2 expressing vector by using JetPrime (PolyPlus Transfection, Illkirch, France). 48 h after transfection, protein and RNA expression was evaluated.

For treatment with the proteasome inhibitor MG132 (Sigma-Aldrich), cells were seeded, after 24 h medium was replaced with medium containing 10 µM MG132 and after 6 h cells were collected. Melanoma Cultured Medium (CM) was taken from sub-confluent dishes of melanoma cells grown in RPMI/10%FBS medium, then the medium was changed to serum-free medium.

For co-culture experiments, HFFGFP-labelled and melanoma cells (ratio 1:1) were plated in 6-well culture plates and after 3 days images were acquired by using the light channel of Bio-Rad ZOE fluorescent cell imager. Cells were collected and GFP positive cells were detected by flow cytometric analysis (BD Accuri™ C6, BD Biosciences, Franklin Lakes, NJ, USA).

### Western blotting analysis

Cells were lysed in RIPA buffer in presence of protease and phosphatase inhibitors (Santa Cruz Biotechnology, Santa Cruz, CA, USA). Protein concentrations were determined by colorimetric assay (Pierce™ BCA Protein Assay Kit, Thermo Scientific). Western blotting was performed using 35–40 µg of protein extracts (60 µg for the detection of LATS1 phosphorylation), using the following primary antibodies: α-tubulin (sc- 32293), Bcl-2 (sc-509) and Bcl-xL (sc-8392) were from Santa Cruz Biotechnology YAP (#12395), phosphorylated YAP (S127, #4911), LATS1 (#3477), phosphorylated LATS1 (#8654), MST2 (#3952), MOB1 (#13730), CTGF (connective tissue growth factor, also known as CCN2, #10,095), vinculin (#13901), pERK1/2 (#9106), ERK (#9102) and H3 (#4499) were from Cell Signaling (Danvers, MA, USA); β-actin (#A1978), alpha-smooth muscle actin, α-SMA (#A5228) was from Sigma-Aldrich; heat shock protein (HSP)72/73 (#HSP01) was from Calbiochem (San Diego, CA, USA). Enhanced Chemiluminescent Substrate method (LiteAblotTURBO, Euroclone) was used to detect immunostained bands, except for the detection of phosphorylated LATS1, by Clarity Max Western ECL Substrate (Bio-Rad Laboratories, Hercules, CA, USA). ChemiDoc System instrument (Bio-Rad Laboratories) was used to acquire images, while ImageJ software was used for densitometric evaluation and normalization with relative controls.

### Total RNA extraction and qRT-PCR

Total RNA was extracted using a Qiagen RNeasy Mini kit (Qiagen, Hilden, Germany) according to the manufacturer’s instructions. Reverse transcription was performed using RevertAid Reverse Transcriptase (Thermo Scientific). Reaction conditions were: 50 °C for 60 min, 85 °C for 5 min, 4 °C until stopped. qRT-PCR was performed using a QuantStudio 6 Flex Real-Time PCR System (Applied Biosystems, Foster City, CA, USA), using the SYBR green dye detection method. The mRNA levels were normalized using β-actin. Primers used to analyze each gene are listed in Supplementary Table S1. The results were evaluated by the ΔΔCt method.

### RNA-seq and bioinformatic analyses

Quantity and integrity of the extracted RNA were assessed by NanoDrop Spectrophotometer (NanoDrop Technologies, Wilmington, DE, USA) and by Agilent 2100 Bioanalyzer (Agilent Technologies, Santa Clara, CA, USA), respectively. RNA libraries for sequencing were generated in triplicate using the same amount of RNA for each sample according to the Illumina Stranded Total RNA Prep kit with an initial ribosomal depletion step using Ribo-Zero Plus (Illumina, San Diego, CA, USA). The libraries were quantified by qPCR and sequenced in paired-end mode (2 × 100 bp) with NovaSeq 6000 (Illumina). For each sample generated by the Illumina platform, a pre-process step for quality control was performed to assess sequence data quality and to discard low-quality reads. RNA-seq data were analysed with “rnaseq” version 3.3 pipeline included into the nf-core platform (https://nfco.re/rnaseq) with default parameters [28]. Differential expression analysis was carried out using DESeq2 package [29]. Normalized counts were expressed as the variance stabilizing transformation function. Adjusted p value < 0.05 and a |log2FC|> 1 were defined as the cut-off criteria for up- and downregulated genes. Enrichment analysis and Disease Ontology gene set interpretation were performed with the ShinyGO version 0.76 [30].

### Transwell migration and wound healing assays

For transwell migration assay, 5 × 10^4^ cells were seeded in serum-free media into the upper chamber of 24 Well ThinCert Cell Culture Inserts (Greiner Bio-One S.r.l, Cassina de Pecchi, Italy). The lower well contained medium with 10% FBS. After 6 h incubation at 37 °C, cells remaining on the top side of the membrane were removed, and migrated cells stained, photographed, and counted. From each transwell, several images were acquired by using the light channel of Bio-Rad ZOE fluorescent cell imager (Bio-Rad Laboratories).

For wound healing assay, 1.8 × 10^3^ melanoma or HFF cells were seeded in Culture-Insert 2 Well in µ-Dish 35 mm (ibidi GmbH, Gräfelfing, Germany). After 24 h, septa were removed and the images relative to starting point (T0) were acquired with Nikon Eclipse Ts100 phase-contrast microscope at ×4 magnification. The closure of the septa caused by the migrating melanoma cells was evaluated by acquiring images after 6 h and the distance between the two cell fronts was measured. As regarding HFF cells, after 24 h medium was replaced with CM derived from 24 h of melanoma cultured cells, septa were removed and the images were acquired to evaluate the closure of the septa. In all experiments, the CM used for stimulating HFF was normalized to the number of melanoma adherent cells.

### ELISA

The level of secreted CTGF by melanoma cells was assayed by ELISA kit according to the manufacturer’s instructions (MyBiosource, Inc, San Diego, CA, USA) normalising the supernatants to the number of adherent cells.

### Cell viability in different stiffness condition

Melanoma cells were seeded on 6-well plates coated with Hydrogels bound with type I collagen from bovine skin or rat tail at different elastic modulus (kPA) (Softwell, Cell Guidance Systems, Cambridge, UK). After 48 h from seeding or from transfection, images were acquired by using the light channel of Bio-Rad ZOE fluorescent cell imager, cells were detached, stained with Trypan Blue solution and both viable cells excluding the dye, and nonviable cells absorbing the dye and appearing blue were counted by CellDrop automated cell counter (DeNovix, Wilmington, DE, USA).

### Analysis of cell proliferation/viability

Melanoma cells were seeded at a density of 3000 cells/well in 96-well plates and treated for 24 h with increasing concentration of Verteporfin (MedChemExpress, Sollentuna, Sweden), a specific YAP inhibitor [31]. Proliferation/viability was evaluated by measuring 3-[4,5-dimethylthiazol-2-yl]-2,5-diphenyltetrazolium bromide inner salt (MTT, Sigma-Aldrich) dye absorbance as previously reported [32].

### Statistical analysis

Results are expressed as mean ± standard deviation (or standard error of mean when indicated) of at least three independent experiments, unless specified. Differences between groups were analysed with an unpaired two-tailed student’s *t* test and considered statistically significant for *p* < 0.05.

## Results

### RNAseq analysis highlights a Hippo pathway linked signature in silenced Bcl-2 melanoma cells

**Fig. 1:**
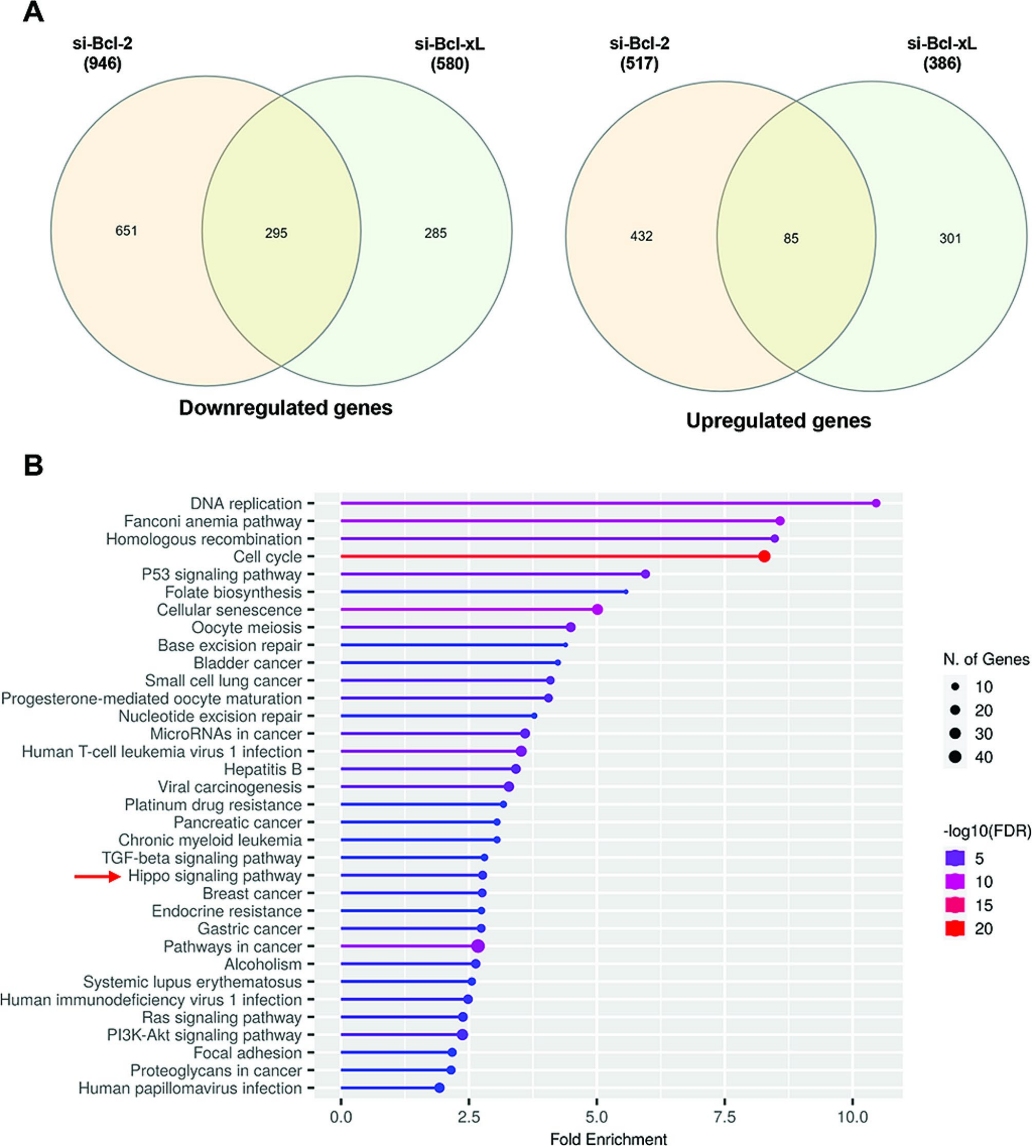
Transcriptional program in Bcl-2 and Bcl-xL silenced A375 cells determined by RNA-seq analysis. **A** Venn diagram of the overlap between significantly differentially expressed genes (either positively or negatively) upon transfection with either si-Bcl-2 or si-Bcl-xL compared with control, from three biological repeats. **B** Top enriched GO terms of Bcl-2-downregulated genes in A375 cells, as determined by KEGG database

In order to deeper characterize the specific functions of Bcl-2, we performed RNA-seq analysis after siRNA-mediated transient knockdown of either Bcl-2 (si-Bcl-2) or Bcl-xL (si-Bcl-xL) in human melanoma A375 cell line (protein and mRNA knockdown validation in Supplementary Figure S1). Differentially expressed genes found in si-Bcl-2 or si-Bcl-xL compared to their relative controls are listed in Supplementary Table S2 and S3, respectively. As expected, a subset of common genes (380) was impacted similarly by depletion of either Bcl-2 or Bcl-xL (Supplementary Table S4), while expression of a larger number of genes was distinctly affected in a protein-specific manner. Indeed, 651 and 432 genes were significantly downregulated or upregulated, respectively, at least two-fold upon Bcl-2 depletion, while not significantly affected by Bcl-xL depletion (Fig. 1A). Conversely, expression of 586 other genes was significantly affected by Bcl-xL depletion, but not by Bcl-2 modulation (Fig. 1A). The sets of genes preferentially regulated by Bcl-2 (Bcl-2-regulated genes) or Bcl-xL (Bcl-xL-regulated genes) were further analysed on the basis of gene ontology (GO) enrichment using the KEGG database. Not significant upregulated GO enriched pathways were found, while a varied list of downregulated GO was enriched by KEGG in si-Bcl-2 condition (Fig. 1B). Among them there were pathways not specifically associated with cancer or associated to tumor histotypes different from melanoma. GO also listed pathways already known to be linked to Bcl-2 function such as Cell Cycle, MicroRNA in Cancer, Resistance to Drugs, Ras [33] and PI3-Akt Pathways [7]. Interestingly, among the downregulated GO pathways we found the Hippo pathway, which was not significantly modulated in si-Bcl-xL condition KEGG analysis (Supplementary Figure S2) and for which few data have been reported to date regarding the link to Bcl-2. The KEGG gene set associated to the Hippo pathway contains the following genes downregulated in si-Bcl-2 condition: NF2 (neurofibromin 2, also known as Merlin), TEAD4, TP73, CCND3 (Cyclin D3), TEAD2, BIRC5 (survivin), SOX2, MYC TGFB2, BMP2, BMP6, FZD1, FZD4, FZD5 and YWHAZ.

### Bcl-2 affects the Hippo pathway in cancer cells

**Fig. 2:**
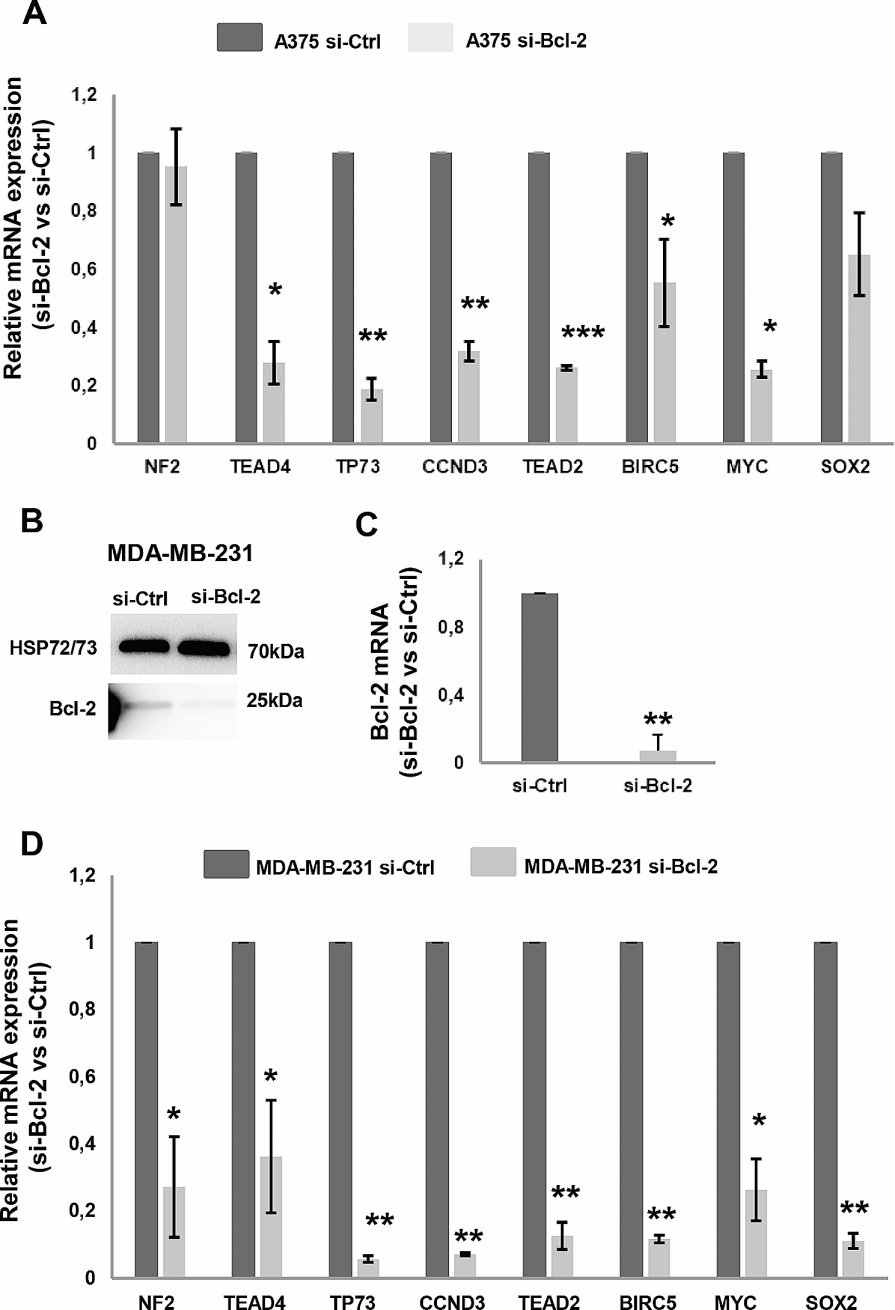
Validation of genes modulated by Bcl-2, enriched by KEGG and associated to Hippo pathway. **A, D** qRT-PCR analysis of NF2, TEAD4, TP73, CCND3, TEAD2, BIRC5, MYC and SOX2 mRNA expression in A375 (A) and MDA-MB-231 (D) cells transiently transfected with siRNA SMARTpools targeting Bcl-2 (si-Bcl-2) or with control siRNA (si-Ctrl). si-Bcl-2 respect to si-Ctrl is reported. **B** Western blot analysis of Bcl-2 protein expression and **C** qRT-PCR analysis of Bcl-2 mRNA level in MDA-MB-231. Representative images of three independent experiments with similar results. HSP72/73 was used to check equal loading and transfer. **A, C, D** Data represent ratio (mean ± standard error of mean) of mRNA expression normalized to β-actin gene in silenced cell condition versus controls from three independent biological repeats (*n* = 3), except for BIRC5 and SOX2 in A (*n* = 5) and TEAD4 in D (*n* = 4). Statistical analysis was performed applying unpaired two-tailed student’s t test *p < 0.05, **p < 0.01, ***p < 0.001

Several selected genes from Hippo pathway were validated by RT-qPCR in independent experiments of transient Bcl-2 silencing in A375 cells, confirming the downregulation of TEAD4, TP73, CCND3, EAD2, and MYC (Fig. 2A).

In order to generalize these results, the modulation of selected genes from Hippo pathway after Bcl-2 silencing was also determined in human breast adenocarcinoma, MDA-MB-231, (Fig. 2B-D) and human non-small cell lung cancer, H460, cell lines (Supplementary Figure S3). In MDA-MB-231 cells, all the tested genes were significantly downregulated in si-Bcl-2 condition, except for TEAD4, while only for TEAD2 transcript a significant modulation was observed in Bcl-2 silenced H460 cells (Supplementary Fig. S3), suggesting that Bcl-2 can affect Hippo pathway depending on the tumor histotype.

**Fig. 3:**
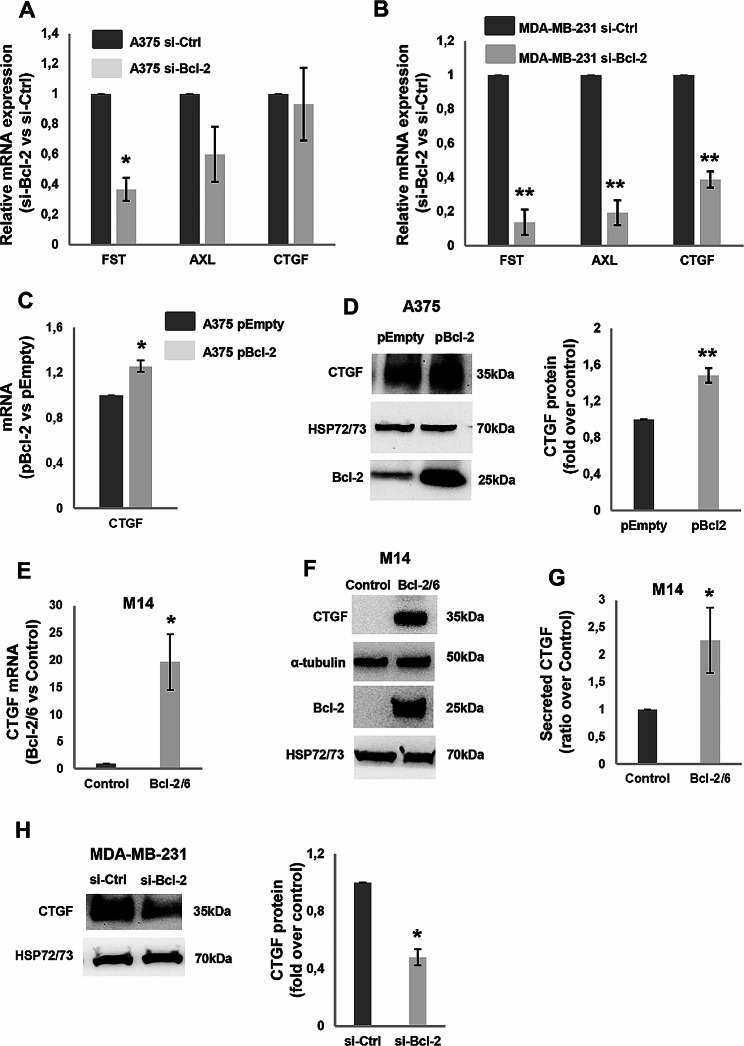
Modulation by Bcl-2 of YAP target genes. **A, B** qRT-PCR analysis of FST, AXL and CTGF mRNA expression in A375 (A) and MDA-MB-231 (B) cells transiently transfected with siRNA SMARTpools targeting Bcl-2 (si-Bcl-2) or with control siRNA (si-Ctrl). si-Bcl-2 respect to si-Ctrl is reported. **C** qRT-PCR and **D** Western blot analyses with relative densitometric analysis of CTGF expression in A375 cells transiently transfected with empty (A375 pEmpty) or Bcl-2 (A375 pBcl-2) expressing vectors. Bcl-2 protein expression was evaluated to validate overexpression efficiency. **E** qRT-PCR and **F** Western blot analyses of CTGF expression evaluated in control (Control) and Bcl-2 overexpressing (Bcl-2/6) M14 clones. The expression level of Bcl-2 protein in the stable clones was also verified. **G** ELISA of CTGF levels in conditioned medium (CM) derived from M14 Control and Bcl-2 overexpressing cells. CTGF levels were normalized to the number of adherent cells and expressed as ratio (mean ± standard deviation) of M14 Bcl-2/6 clone over control. **H** Western blot analysis of CTGF protein expression with relative densitometric analysis in MDA-MB-231 si-Bcl-2 and si-Ctrl samples. **A-C, E** Data represent ratio (mean ± standard error of mean in A and B, mean ± standard deviation in C and E) of mRNA expression normalized to β-actin gene in silenced cell condition versus controls (A, B) or Bcl-2 overexpressing cells versus control (C, E), from three independent biological repeats, except for AXL and CTGF in A (*n* = 5). **D, F, H** Representative images of three independent experiments with similar results. HSP72/73 and α-tubulin were used to check equal loading and transfer. **A-H** Statistical analysis was performed applying unpaired two-tailed student’s t test, *p < 0.05, **p < 0.01

We also assessed the expression of other known YAP target genes, such as FST (also known as Follistatin), AXL, and CTGF [23, 34], not included in differential gene expression and ontology. FST gene expression was found decreased in A375 (Fig. 3A), MDA-MB-231 (Fig. 3B) and H460 (Supplementary Figure S3) cells after Bcl-2 depletion. RNAseq analysis in A375 si-Bcl-2 condition confirmed a significant FST gene expression reduction (log2Fold change= -1,65; *p-value* 4,11 × 10^7^). A significant decrease of AXL transcript was found upon Bcl-2 silencing in MDA-MB-231 cells (Fig. 3B), while a non-significant downregulation and no modulation was observed, respectively, in A375 (Fig. 3A) and H460 (Supplementary Figure S3) cells. A not significant decrement of AXL transcript was found by RNAseq analysis in A375 si-Bcl-2 condition (log2Foldchange = 0.43; *p-value* = 0.088).

When the Bcl-2 protein was overexpressed in A375 cells after transient transfection with a plasmid coding for the corresponding sequence (pBcl-2), a significant increment of both mRNA (Fig. 3C) and protein of CTGF was observed (Fig. 3D), respect to cells transfected with the corresponding empty vector (pEmpty). We validated these results in human melanoma M14 clone stably overexpressing Bcl-2. As reported in Fig. 3, M14 cells forced to express Bcl-2 (M14 Bcl2/6) showed increased expression of CTGF transcript (Fig. 3E) and intracellular protein (Fig. 3F) and higher level of secreted CTGF in the conditioned medium (Fig. 3G). The Bcl-2-dependent modulation of CTGF was also found in MDA-MB-231 cell line, where Bcl-2 silencing decreased the expression of both CTGF transcript (Fig. 3B) and protein (Fig. 3H) compared to control, while a not significant CTGF protein perturbation was observed in H460 cells (data not shown) after Bcl-2 genetic modulation.

**Fig. 4:**
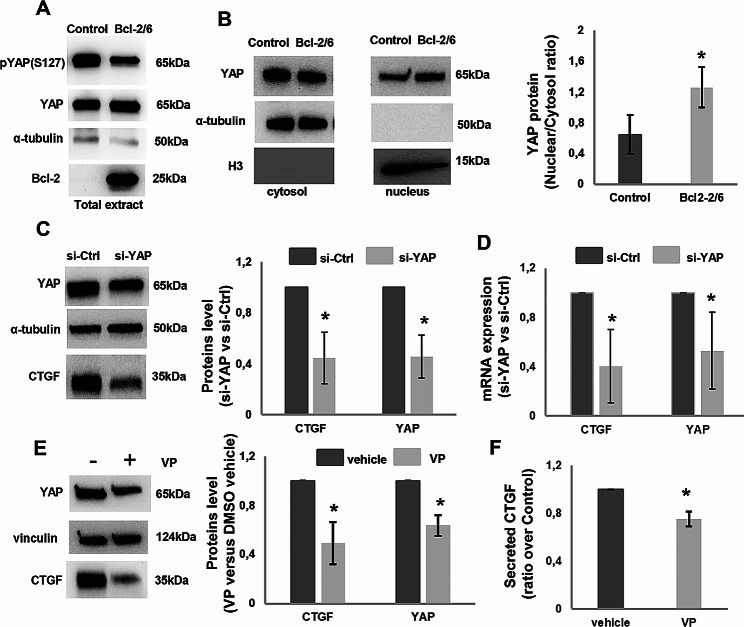
Modulation of CTGF expression by Bcl-2 through YAP in M14 cells. **A** Western blot analysis of YAP phosphorylation, pYAP (S127), and YAP protein in total protein extracts of control (Control) and Bcl-2 overexpressing (Bcl-2/6) clones. **B**) Western blot with relative densitometric analysis expressed as “nuclear/cytosol ratio” of YAP protein expression in the nuclear (nucleus) and cytoplasmic (cytosol) extracts of Control and Bcl-2/6 clones. α-tubulin and histone H3 were used as cytoplasmic and nuclear loading control, respectively. One representative western blot analysis out of three with similar results is reported. **C** Western blot with relative densitometric analysis and **D** qRT-PCR analyses of YAP and CTGF expression in Bcl-2/6 cells transiently transfected with siRNA SMARTpools targeting YAP (si-YAP) or with control siRNA (si-Ctrl). Data represent ratio (mean ± standard deviation) of mRNA expression normalized to β-actin in silenced cell condition versus controls from three independent biological repeats. **E** Western blot and densitometric analyses of YAP and CTGF proteins expression in Bcl-2/6 cells in the presence or absence of Verteporfin, VP (1 µM, 24 h). **A-C, E** Representative images of three independent experiments with similar results. α-tubulin and vinculin are shown as loading and transferring control. **F** ELISA analysis of CTGF protein levels in Conditioned Medium (CM) derived from Bcl-2/6 cells treated with VP. CTGF levels were normalized to the number of adherent cells and expressed as ratio (mean ± standard deviation) of treated cells over control in three independent experiments. **B-F** Statistical analysis was performed applying unpaired two-tailed student’s t test, *p < 0.05

Although the analysis of YAP target genes expression is recognized as a robust and quantifiable method to assess YAP activity [23], the level of YAP phosphorylation and nuclear translocation in melanoma cells overexpressing Bcl-2 was also assessed. Western blotting analysis of total protein extracts showed a reduced level of YAP phosphorylation in M14 cells overexpressing Bcl-2 (Fig. 4A), while the level of the total YAP protein was not affected, suggesting an activation of YAP. In accordance with this result, western blotting analysis of nuclear and cytoplasmic fractions showed an increased nuclear localisation of YAP protein in Bcl-2 overexpressing M14 cells respect to control (Fig. 4B). The inhibition of YAP expression obtained by transfecting Bcl-2 overexpressing cells with specific siRNA (M14 Bcl-2/6 si-YAP), able to reduce both YAP protein (Fig. 4C) and mRNA expression (Fig. 4D), resulted in the downregulation of CTGF, at both protein and transcript level (Fig. 4C, D). In order to validate this result, we also applied the commonly used YAP inhibitor Verteporfin, disrupting the YAP-TEAD interaction [31]. Treatment with Verteporfin (1 µM, 24 h) was sufficient to inhibit both YAP and CTGF protein expression (Fig. 4E) and CTGF secretion (Fig. 4F) respect to control, while cell viability was not significantly affected (Supplementary Figure S4).

### Bcl-2 regulates the Hippo pathway core proteins in cancer cells

**Fig. 5:**
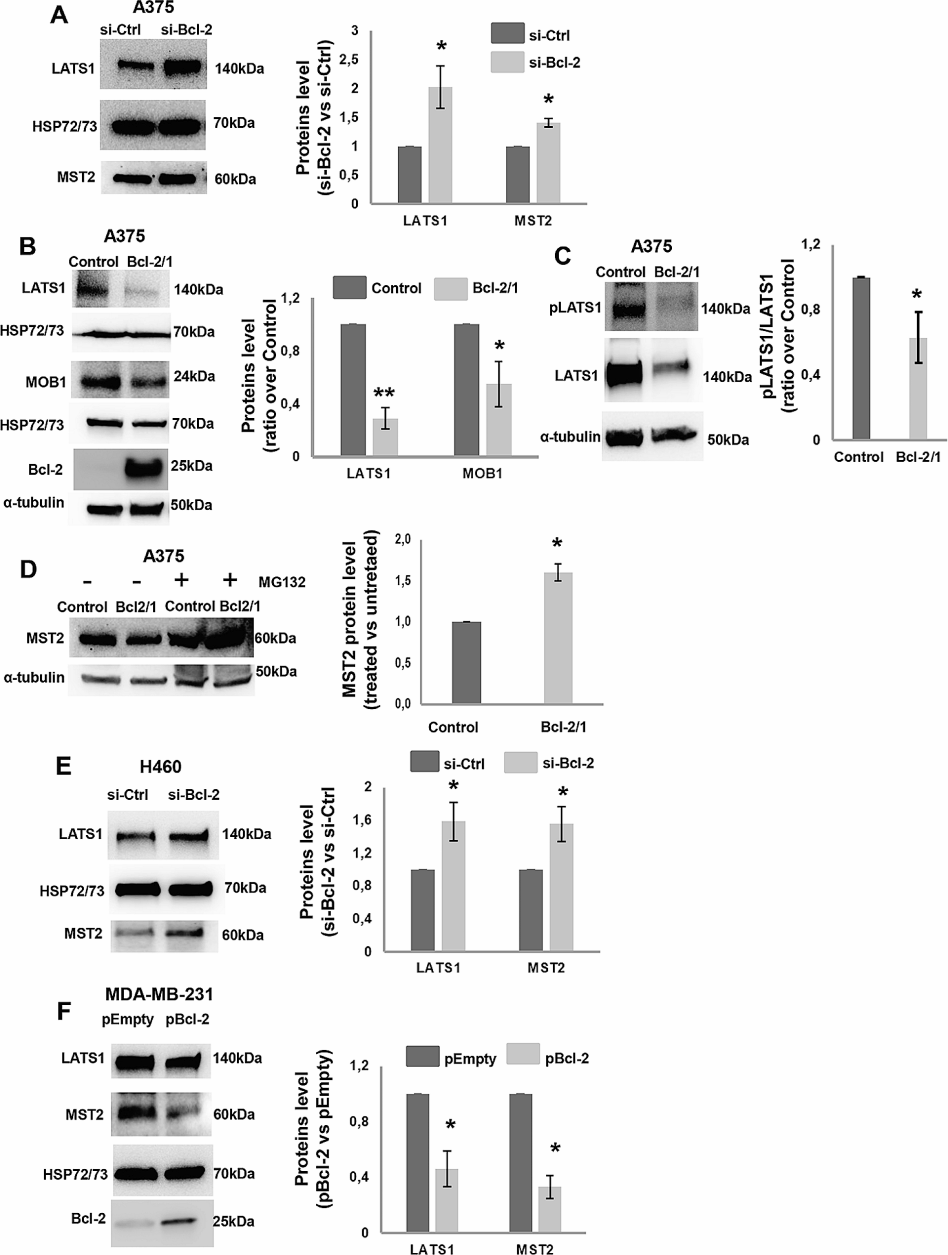
Regulation of Hippo pathway core proteins by Bcl-2 in cancer cells. **A, B** Western blot and relative densitometric analysis of LATS1 and MST2 proteins in **A** A375 cells transiently transfected with siRNA targeting Bcl-2 (si-Bcl-2) or control siRNA (si-Ctrl) and of LATS1, MST2 and MOB1 proteins in **B** A375 control (Control) and Bcl-2 overexpressing (Bcl-2/1) stable clones. The expression level of Bcl-2 protein in the stable clones was also verified. **C** Western blotting and densitometric analyses of phosphorylated LATS1 (pLATS1) and total LATS1 in A375 control and Bcl-2 overexpressing (Bcl-2/1) cells. **D** Western blot analysis of MST2 and relative densitometry from A375 control and Bcl-2 overexpressing (Bcl-2/1) cells in the presence or absence of MG132. **E, F** Western blot and relative densitometric analysis of LATS1 and MST2 proteins in **E** H460 cell s transiently transfected with siRNA targeting Bcl-2 (si-Bcl-2) or with control siRNA (si-Ctrl) and **F** in MDA-MB-231 cells transiently transfected with empty (pEmpty) or Bcl-2 (pBcl-2) expressing vectors. Bcl-2 protein expression was evaluated to validate overexpression efficiency. **A-F** One representative western blot analysis out of three with similar results is reported. HSP72/73 and α-tubulin were shown as loading and transferring control. Data represent ratio (mean ± standard deviation) of protein density normalized to HSP72/73 or α-tubulin in silenced or overexpressing cell conditions versus controls from three independent biological repeats. Statistical analysis was performed applying unpaired two-tailed student’s t test, *p < 0.05, **p < 0.01

After having validated the Hippo pathway signature and having demonstrated the YAP-mediated regulation by Bcl-2 protein levels, we investigated the molecular mechanism analysing whether Bcl-2 was able to affect the expression of proteins acting upstream of YAP in the Hippo pathway: LATS1 and MST2. As displayed in Fig. 5, transient knockdown of Bcl-2 in A375 cells (Fig. 5A) increased the level of both LATS1 and MST2 proteins. These results were further confirmed in A375 Bcl-2/1 clone stably overexpressing Bcl-2, showing reduced level of both LATS1 protein (Fig. 5B) and phosphorylation (Fig. 5C) when compared to control ones. Cells forced to express Bcl-2, also showed reduced level of MST2 protein compared to control (Fig. 5D).

Transient knock-down of Bcl-2 increased the level of both LATS1 and MST2 proteins also in H460 cells (Fig. 5E), while in MDA-MB-231 cells a significant reduction of both LATS1 and MST2 was observed after the transient Bcl-2 overexpression (Fig. 5F). These data suggested that Bcl-2 could regulate YAP activity by affecting the cellular availability of the main YAP upstream regulators.

To evaluate a possible involvement of Bcl-2 on MST2 protein stabilization, as putative mechanism of regulation, which in turn may positively affects the YAP activity, the effect of the proteasome inhibitor, MG132, on MST2 protein level was evaluated in relation to different level of Bcl-2 protein. As reported in Fig. 5D, in the presence of MG132, the increment of MST2 protein was higher in Bcl-2 overexpressing cells respect to control, thus indicating a major portion of MST2 protein prone to be eliminated by proteasome after Bcl-2 overexpression, resulting in less LATS1 activation in Bcl-2 overexpressing A375 cells and increased YAP activation. Results regarding MST2 protein level after proteasome inhibition were confirmed in Bcl-2 overexpressing M14 cells (Supplementary Fig S5). Interestingly, in both melanoma models, also MOB1 was found decreased after Bcl-2 overexpression (Fig. 5B and Supplementary Fig. S5).

### Functional implications of bcl-2-mediated regulation of YAP in melanoma cells

**Fig. 6:**
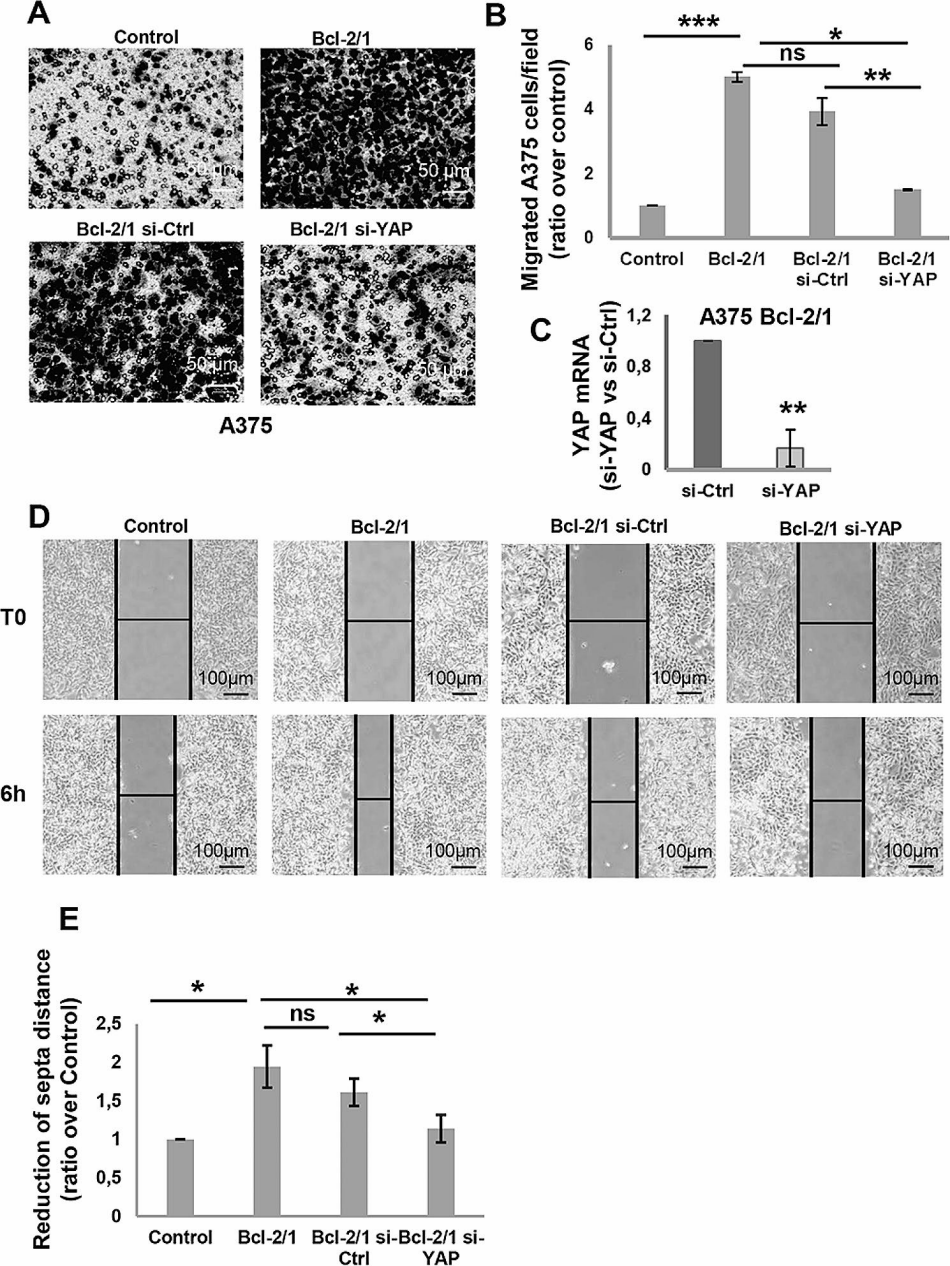
Modulation of in vitro cell migration by YAP silencing in Bcl-2 overexpressing melanoma clones. **A** Representative images and **B** relative quantification of in vitro cell migration of control (Control) and Bcl-2 overexpressing A375 (Bcl-2/1) clones and Bcl-2/1 clone transiently transfected with si-YAP (Bcl-2/1 si-YAP) or with control siRNA (Bcl-2/1 si-Ctrl). The values are reported as ratio (mean ± standard deviation) of number of migrated cells/field versus control. The quantification was performed by counting the number of migrated cells in at least 5 fields for each condition. **C** qRT-PCR analysis to evaluate efficiency of YAP silencing in A375 Bcl-2/1 cells transiently transfected with siRNA SMARTpools targeting YAP (si-YAP) or with control siRNA (si-Ctrl). **D** Representative images and **E** relative quantification of in vitro ability of A375 control and A375 Bcl-2/1 clones and A375 Bcl-2/1 clone transiently transfected with si-YAP or with control si-Ctrl cells to migrate in wound healing assay. The values are reported as ratio (mean ± standard deviation) respect to control. **A-E** Experiments have been conducted in biological triplicate, except for B (biological duplicate is reported). Statistical analysis was performed applying unpaired two-tailed student’s t test, *p < 0.05, **p < 0.01, ***p < 0.01, #*p* = 0.05, ns = not significant

We next investigated the implication of YAP in cellular processes that are known to be regulated by Bcl-2, such as cell migration. In accordance with previously published data [7], stable Bcl-2 overexpression induced a significant increase of in vitro cell migratory capacity of melanoma cells (Fig. 6A, B and Supplementary Fig S6). The ability of YAP to mediate this Bcl-2 property was demonstrated in Bcl-2 overexpressing A375 and M14 cells transiently transfected with specific small interference siRNA smart pool targeting YAP (si-YAP), where the downregulation of YAP significantly decreased the ability of A375 (Fig. 6A-E) and M14 cells (Supplementary Fig S6) to migrate compared to cells transfected with the non-targeting siRNA in both transwell migration and wound healing assays.

**Fig. 7:**
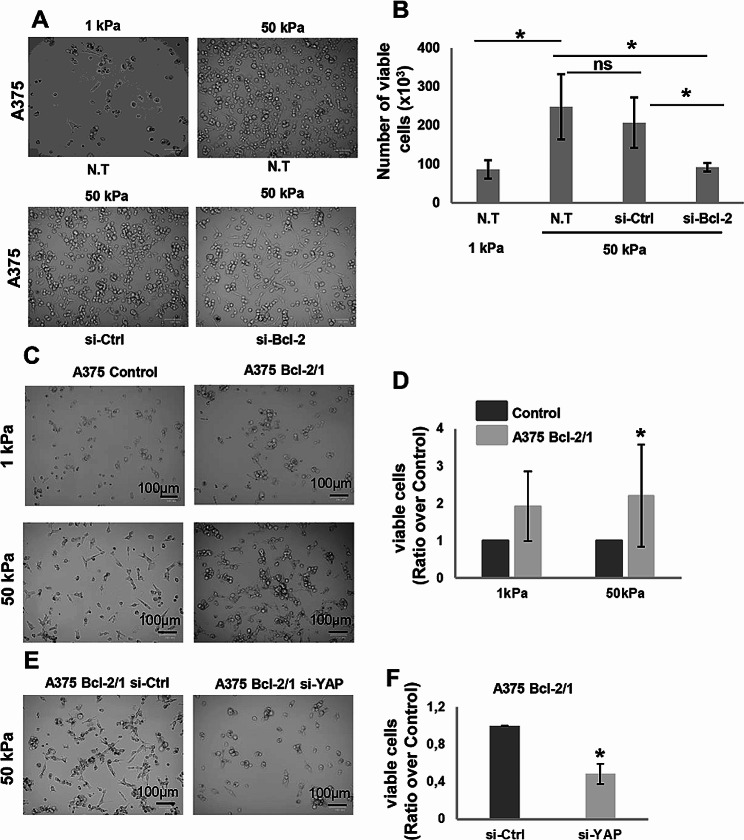
Regulation of melanoma cell viability by Bcl-2 in different stiffness conditions. **A** Representative images and **B** relative quantification of viability of A375 cells plated on different stiff conditions, 1 kPa and 50 kPa, and of A375 cells plated on 50 kPa and transiently transfected with siRNA SMARTpools targeting Bcl-2 (si-Bcl-2) or with control siRNA (si-Ctrl). The values are reported as number (mean ± standard deviation) of viable cells. **C** Representative images and **D** relative quantification of viability of A375 Control and Bcl-2 overexpressing (A375 Bcl-2/1) clones seeded on 1 kPa and 50 kPa stiffness. The values are reported as ratio (mean ± standard deviation) of viable A375 Bcl-2/1 cells over control. **E** Representative images and **F** relative quantification of viability of A375 Bcl-2/1 cells seeded on 50 kPa stiffness and transiently transfected with siRNA SMARTpools targeting YAP (si-YAP) or with control siRNA (si-Ctrl). The values are reported as ratio (mean ± standard deviation) of viable cells over control. **B, D, F** Cell viability was assessed by Trypan blue staining and using an automatic cell counter. **A-F** Experiments have been performed in three biological replicates. Statistical analysis was performed applying unpaired two-tailed student’s t test, *p < 0.05; ns = not significant

As Hippo pathway is well-known to be linked to alterations of extracellular matrix (ECM) rigidity [35], we investigated whether Bcl-2 affects the response of melanoma cells to different stiffness conditions and whether YAP mediates this response. In order to assess the impact of Bcl-2 on the behaviour of melanoma cells in response to different mechanical properties of collagen, we used collagen-coated hydrogels plate. In particular, we exposed melanoma cells to collagen with different degree of stiffness, such as 1 kPa resembling normal tissue, or 50 kPa, representing a slightly stiffer environment similar to what is found in tumours [36]. According to published data [36], A375 cells plated on 50 kPa gel showed increased viability than cells seeded in lower stiff conditions, 1 kPa, due to their nature of aggressive solid tumor cells (Fig. 7A, B). Interestingly, Bcl-2 silencing abrogated the ability of A375 cells to growth in 50 kPa stiffness condition, suggesting a role played by Bcl-2 in regulating the mechanobiology of melanoma cells in response to different tumor environmental properties (Fig. 7A, B). This evidence was confirmed when A375 (Fig. 7C-F) and M14 (Supplementary Fig S6) clones stably overexpressing Bcl-2 were plated on different stiffness gels: increased cell viability respect to control was observed when Bcl-2 overexpressing melanoma cells were plated in both 1 kPa and 50 kPa (Fig. 7C, D, Supplementary Fig. 5C, D). Moreover, YAP silencing decreased cell viability of Bcl-2 overexpressing clone plated on 50 kPa gel (Fig. 7E, F, Supplementary Fig S6), indicating the involvement of YAP in the ability of Bcl-2 to promote cell viability in higher stiffness condition.

### Melanoma-specific Bcl-2 promotes fibroblast activation

**Fig. 8:**
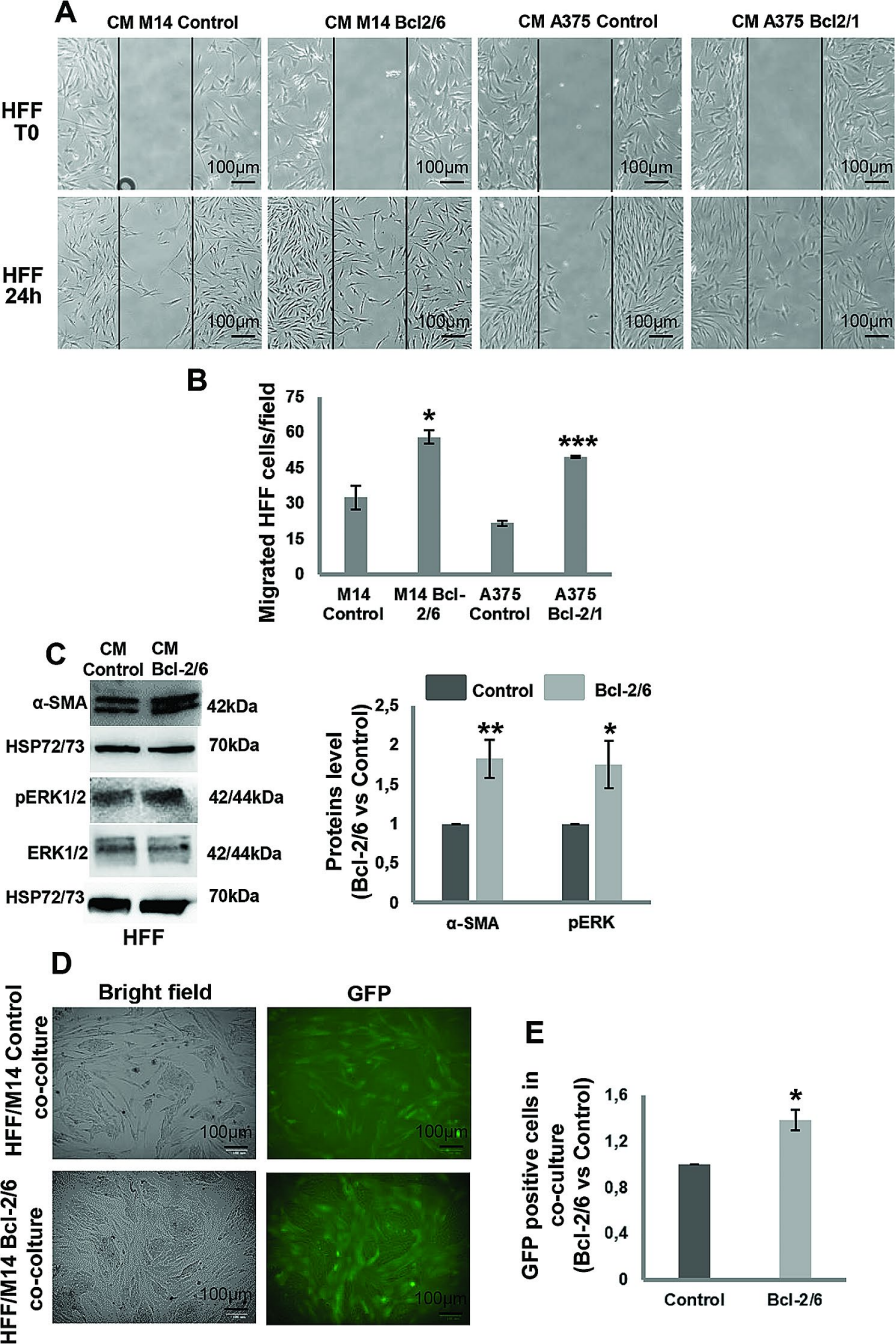
Fibroblast activation by Cultured Medium (CM) from melanoma cells in a Bcl-2-dependent manner. **A** Representative images and **B** quantification of human foreskin fibroblasts (HFF) migration in response to CM from control (CM M14 Control) or Bcl-2 overexpressing (CM M14 Bcl-2/6) M14 clones and control (CM A375 Control) or Bcl-2 overexpressing (CM A375 Bcl-2/1) A375 clones. The values are reported as ratio (mean ± standard deviation) of migrated cells/field respect to control. The quantification was performed by counting the number of migrated cells in at least 3 fields for each condition. **C** Western blot and relative densitometric analysis of α-SMA, pERK1/2 and ERK1/2 proteins in HFF stimulated for 24 h with CM from Control or Bcl-2/6 M14 clones. One representative western blot analysis out of three with similar results is reported. HSP72/73 is shown as loading and transferring control. **D** Representative images and **E** quantification of HFF stably expressing GFP protein in co-culture with M14 Control and M14 Bcl-2/6 cells. The values are reported as ratio (mean ± standard deviation) of GFP positive cells in HFF/M14 Bcl-2/6 cells co-culture respect to HFF/M14 control cells co-culture. **A-E** Experiments have been conducted in triplicates. Statistical analysis was performed applying unpaired two-tailed student’s t test, *p < 0.05, **p < 0.01, ***p < 0.001

**Fig. 9:**
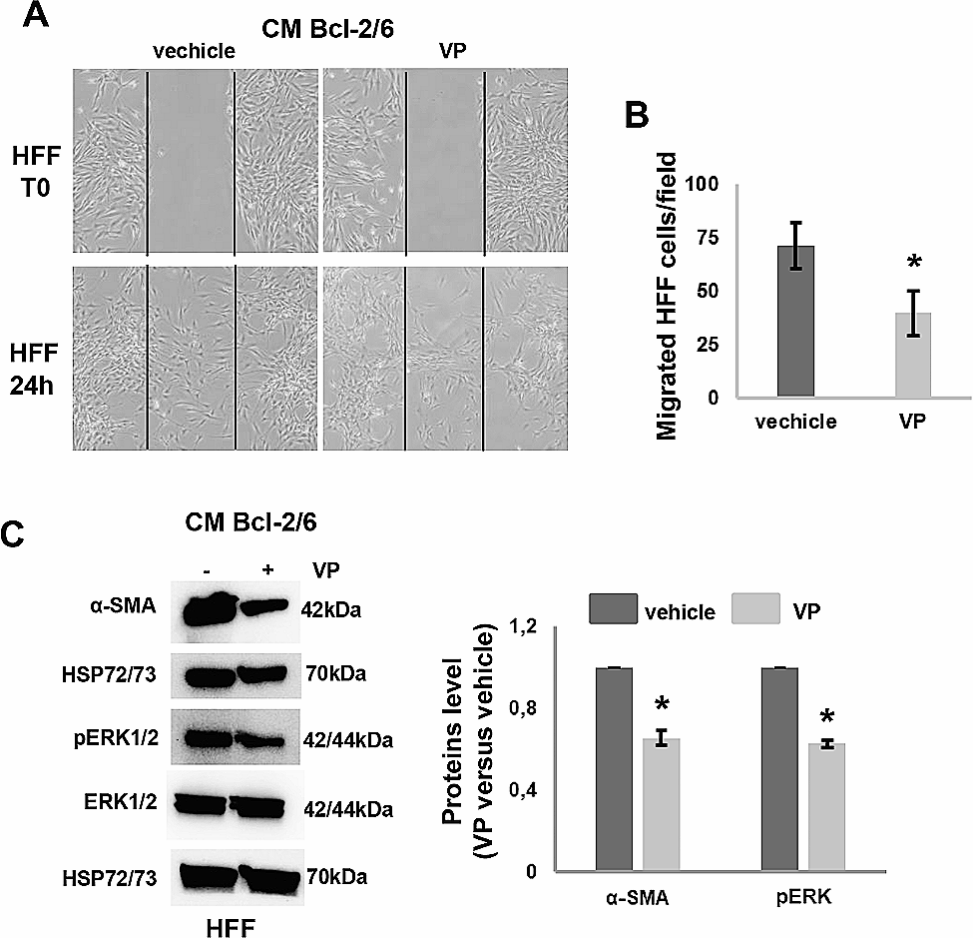
YAP inhibition by Verteporfin reverts fibroblast activation. **A** Representative images and **B** quantification of human foreskin fibroblasts (HFF) migration in response to Conditioned Medium (CM) from Bcl-2 overexpressing M14 (Bcl-2/6 M14) cells treated with Verteporfin (VP, 1µM, 24 h) or vehicle. The values are reported as ratio (mean ± standard deviation) of migrated cells/field respect to control. The quantification was performed by counting the number of migrated cells in at least 3 fields for each condition. **C** Western blot and densitometric analyses of α-SMA, pERK1/2 and total ERK1/2 proteins in HFF stimulated for 24 h with CM from Bcl-2/6 M14 cells in presence or absence of VP. One representative western blot analysis out of two with similar results is reported. HSP72/73 is shown as loading and transferring control. **A-C** Experiments have been conducted in triplicates. Statistical analysis was performed applying unpaired two-tailed student’s t test, *p < 0.05

On the basis of these results evidencing the Bcl-2 regulation of Hippo pathway and stiffness, and considering our previous data demonstrating the involvement of melanoma-specific Bcl-2 in the TME, we investigated whether Bcl-2 could promote fibroblast activation. Firstly, we evaluated the ability of Bcl-2 overexpressing melanoma cells to affect in vitro ability of fibroblasts to migrate. As reported in Fig. 8, human foreskin fibroblasts, HFF, exposed for 24 h to CM derived from Bcl-2 overexpressing A375 and M14 clones showed higher capacity to migrate compared to fibroblasts exposed to CM derived from control melanoma cells, evaluated in a wound healing assay (Fig. 8A, B). HFF stably expressing GFP protein, exposed to CM from Bcl-2 overexpressing M14 cells, also expressed higher levels of α-smooth muscle actin (α-SMA), an established cancer associated fibroblasts (CAFs) marker [37], and ERK1/2 phosphorylation (Fig. 8C). Moreover, the percentage of GFP positive cells was higher in HFF/Bcl-2 overexpressing M14 cells co-culture respect to HFF/M14 control cells co-culture (Fig. 8D, E). These results suggest that melanoma-specific Bcl-2 could promote fibroblast activation. In addition, HFF, exposed for 24 h to CM derived from Bcl-2 overexpressing M14 cells treated with Verteporfin (1 µM, 24 h) showed reduced capacity to migrate (Fig. 9A, B) and expressed lower levels of α-SMA and phosphorylated ERK1/2 (Fig. 9C). Thus, indicating that Yap inhibition by Verteporfin is able to revert fibroblast activation induced by cancer-specific Bcl-2.

## Discussion

Although the main function of Bcl-2 and Bcl-xL proteins is anti-apoptotic, their non-canonical role in different tumor histotypes is now generally recognized, being involved in numerous processes, such as autophagy, angiogenesis, cell migration and invasion, regulation of microRNAs [9]. Here, we characterized Bcl-2 functions to a deeper extent by extracting a specific signature by RNAseq analysis after Bcl-2 or Bcl-xL silencing in melanoma models. Interestingly, the regulation by Bcl-2, but not Bcl-xL, of a large number of genes associated with the Hippo pathway, was confirmed in different tumor histotypes, such as breast and lung carcinoma, thus supporting the pleiotropic role of Bcl-2, acting as an important regulator of multiple and diverse cellular processes.

Among the genes regulated by Bcl-2 in both melanoma and breast carcinoma models we identified CTGF, CCND3, TEAD2, FST, MYC and TP73. All these genes, except TEAD2/4, are known target of YAP/TAZ [34], involved in cancer progression and response to therapy. In particular, CTGF is dysregulated in many cancer types, including melanoma [38], breast and lung carcinoma [39, 40], promoting cancer initiation, progression and drug resistance and regulating key properties of the TME [39, 41]. CCND3 is a biomarker component of differentially expressed mRNA signature in melanoma respect to normal tissues [42], and is related to survival outcomes in triple negative breast cancer patients [43]. FST mediates the ability of YAP to stimulate cell invasion in melanoma [23, 44], and its expression correlates with metastasis in a breast cancer mouse model [45] and with survival outcomes in cancer patients [46]. MYC is involved in tumor progression [47] and in the response to therapy of both melanoma [48] and breast carcinoma [49]. TP73 plays a role in the progression of melanoma [50], and breast carcinoma [51], and in YAP-mediated response of breast cancer to therapy [52]. Depletion of TEAD2 suppresses the ability of YAP to induce invasion in melanoma cells [23] while is overexpressed in trastuzumab resistant breast cancer cells [53].

Also, TEAD4 transcript level was found decreased after Bcl-2 depletion in melanoma. It mediates the YAP ability to induce melanoma cell invasion [23], and a genetic variant negatively correlates with melanoma patients survival [54].

Our data also evidenced that some YAP/TAZ target genes, such as NF2, AXL, SOX2 and BIRC5 [34], were regulated by Bcl-2 specifically in breast carcinoma model.

Interacting directly with LATS1/2, the scaffold protein NF2 regulates the activity of central components of the Hippo pathway [21]. NF2 protein level was found reduced in metastatic breast cancer tissues [55, 56]. AXL overexpression is associated to resistance to chemotherapy and targeted therapies [57, 58]. SOX2 is an oncogene in all breast cancer subtypes [59] and its expression correlates with breast cancer aggressiveness [59]. High activity of BIRC5 has been associated with a poor prognosis and worse survival rates in breast cancer patients [60].

In our study we also found the downregulation of both TEAD2 and FST genes, in lung cancer cells after Bcl-2 silencing. Serum FST levels was found higher in lung cancer patients respect to healthy subjects and in patients with lung benign disease [61], while TEAD2 targeting was responsible of cisplatin sensitization in NSCLC [62]. YAP is overexpressed in 60–70% of NSCLC, and YAP amplification occurs in ∼ 15% of squamous lung cell cancers [34, 63]. Moreover, YAP overexpression correlates with poor clinical prognoses in lung cancer patients and resistance to chemotherapies and target therapies [63, 64]. Since few genes associated to Hippo pathways have been validated in lung cancer cells used in our study, we could hypothesize that in this cellular model the downstream genes modulated by YAP are different from those here investigated.

Mechanistically, we demonstrated that in melanoma, breast and lung cancer models Bcl-2 affects Hippo pathway by regulating the level of MST2, a key YAP upstream regulator. We also showed that Bcl-2 regulates MST2 protein stability. Our results are consistent with published data demonstrating that Bcl-2 inhibits protein levels of MST2 in human embryonic kidney cells by inducing proteasomal degradation, and that inhibition of Bcl-2 restores the level of MST2 kinase, resulting in increased cell death [65]. Also, a recent study described a link between Bcl-2 and YAP [66], demonstrating that TEAD4 binds to the promoter regions of Bcl-2 and that YAP silencing abrogated downregulated Bcl-2 protein in colorectal cancer cells [66]. Moreover, YAP overexpression promotes anoikis resistance in melanoma cells and metastasis in in vivo experiments, through the expressions of downstream genes Bcl-2 and Mcl-1 [67]. Together with our results, these data strongly indicate the existence of a bi-directional regulatory loop in cancer involving Bcl-2 and YAP.

As YAP is required for the invasive and migratory ability of melanoma cells [23] and Hippo pathway activation results in changes of ECM stiffness [35], a property of solid tumor relevant for cancer progression and resistance to treatment [35, 68], we also evaluated whether the Bcl-2/YAP axis was crucial in mediating some cellular processes, such as cell migration and proliferation in response to different stiff condition of culture. Interestingly, we found that YAP silencing abolished the ability of Bcl-2 to increase cell migration and to promote cell proliferation of melanoma cells on higher stiffness condition of culture, resembling stiffer environment found in solid tumours.

Finally, in order to study the role of cancer-specific Bcl-2 in TME, we showed that melanoma cells overexpressing Bcl-2 were able to stimulate in vitro fibroblasts migration and proliferation. Moreover, the increased α-SMA protein level and ERK phosphorylation, observed in fibroblasts exposed to CM from Bcl-2 overexpressing cells are indicative of a Bcl-2 dependent activation of fibroblast versus CAF phenotype. The use of CM from Bcl-2 overexpressing melanoma cells treated with the specific YAP inhibitor Verteporfin, reduced in vitro fibroblasts migration, and both α-SMA protein level and ERK phosphorylation, indicating the involvement of YAP in fibroblast activation by cancer-specific Bcl-2.

In addition to the previous evidence of a crosstalk between melanoma cells expressing Bcl-2 and component of the TME, such as macrophages and endothelial cells, these results demonstrated a further relationship between melanoma specific Bcl-2 and TME through the activation of fibroblasts.

In conclusion, here we demonstrated that Bcl-2 regulates Hippo pathway target genes by acting on core proteins upstream to YAP. In this way, Bcl-2 is able to carry out an array of different functions that favor an aggressive phenotype in melanoma cells by promoting cell migration, cellular adaptation to an environment that recapitulates the matrix of solid tumors, and activation of fibroblast versus CAF phenotype, thus suggesting to further explore the possibility of using Bcl-2 inhibitors to reduce cancer progression. In this content, it is worth mentioning that navitoclax, a Bcl-2/Bcl-xL inhibitor, is under evaluation in phase I/II trial study in combination with dabrafenib and trametinib in patients with BRAF-mutant solid tumors, including melanoma (ClinicalTrials.gov Identifier: NCT01989585), and in triple negative breast cancer patients in combination with the PARP inhibitor olaparib in a phase I study (ClinicalTrials.gov Identifier: NCT05358639), while the Bcl-2 inhibitor, TQB3909, is under evaluation in phase Ib/II study in breast cancer patients (ClinicalTrials.gov Identifier: NCT05775575).

### Electronic supplementary material

Below is the link to the electronic supplementary material.

Supplementary Material 1

Supplementary Material 2

Supplementary Material 3

Supplementary Material 4

Supplementary Material 5

Supplementary Material 6

Supplementary Material 7

## Data Availability

No datasets were generated or analysed during the current study.

## Abbreviations

Bcl-2: B-cell lymphoma 2
YAP Yes: Associated protein 1
LATS1: Large Tumor Suppressor Kinase 1
TME: Tumor microenvironment
VEGF: Vascular endothelial growth factor
IL-8: Interleukin-8
CXCR2: C-X-C motif chemokine receptor 2
TEAD: TEA domain
NSCLC: Non-small cell lung cancer
qRT-PCR: Real-Time Quantitative Reverse Transcription PCR
KEGG: Kyoto Encyclopedia of Genes and Genomes
GO: Gene ontology
CCND3: Cyclin D3
CTGF: Connective tissue growth factor
HFF: Human foreskin fibroblasts
GFP: Green fluorescent protein
