# Supplementary material for: Bcl-2 dependent modulation of Hippo pathway in cancer cells
Source: Cell Commun Signal. 2024 May 16;22:277. doi: 10.1186/s12964-024-01647-1 (PMC11097437; doi:10.1186/s12964-024-01647-1)

Supplementary Figure S1

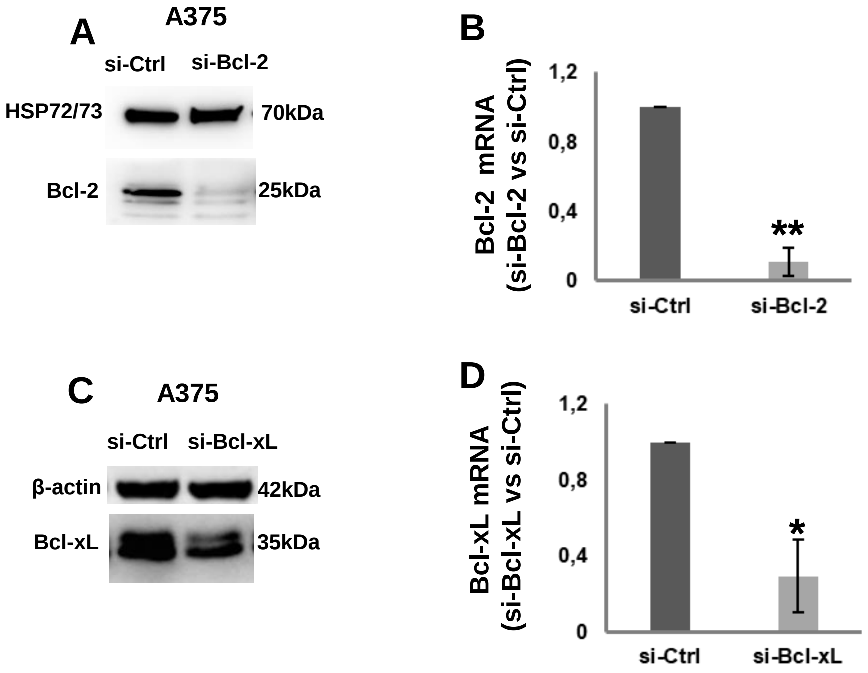

Supplementary Figure S2

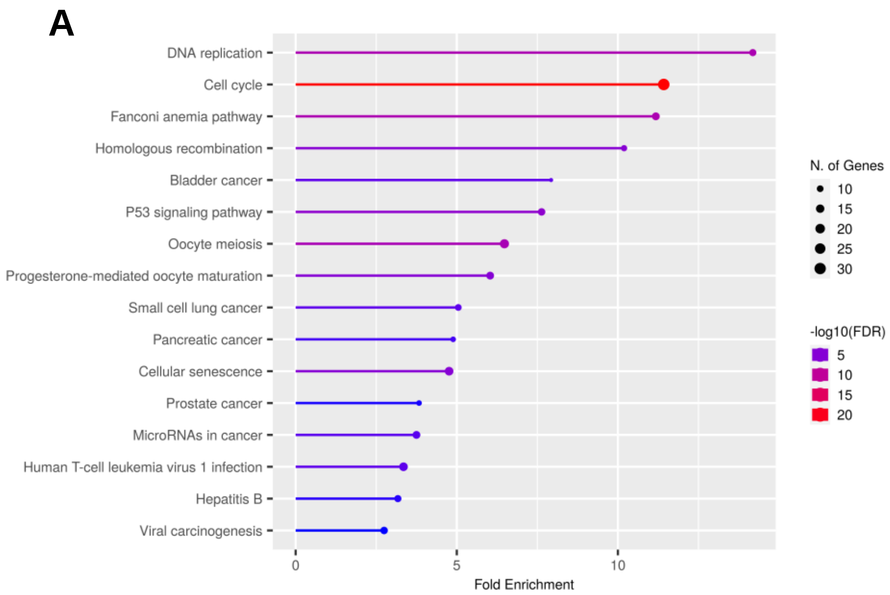

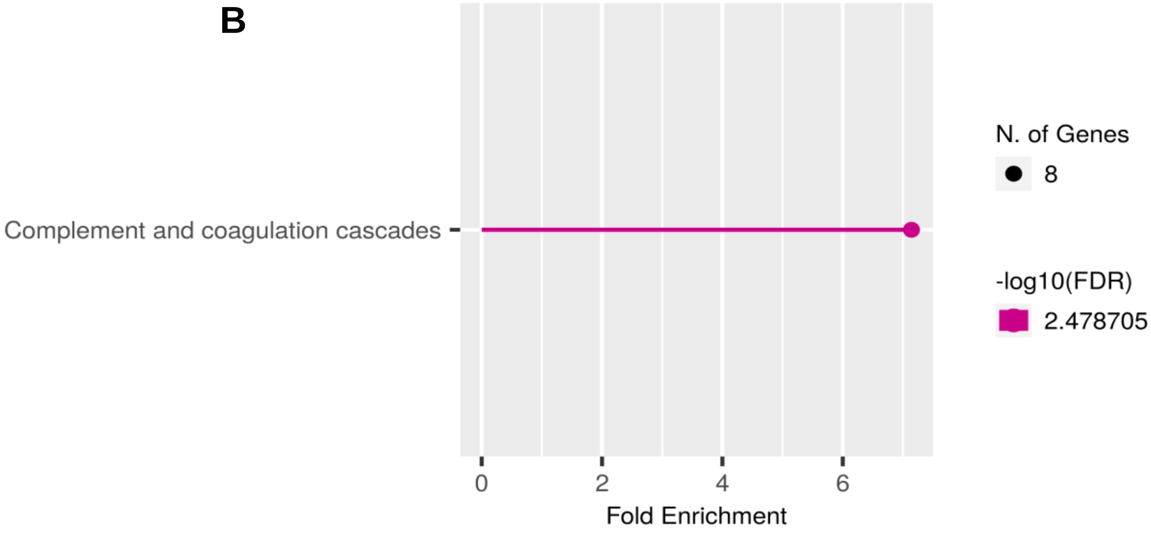

Supplementary Figure S3

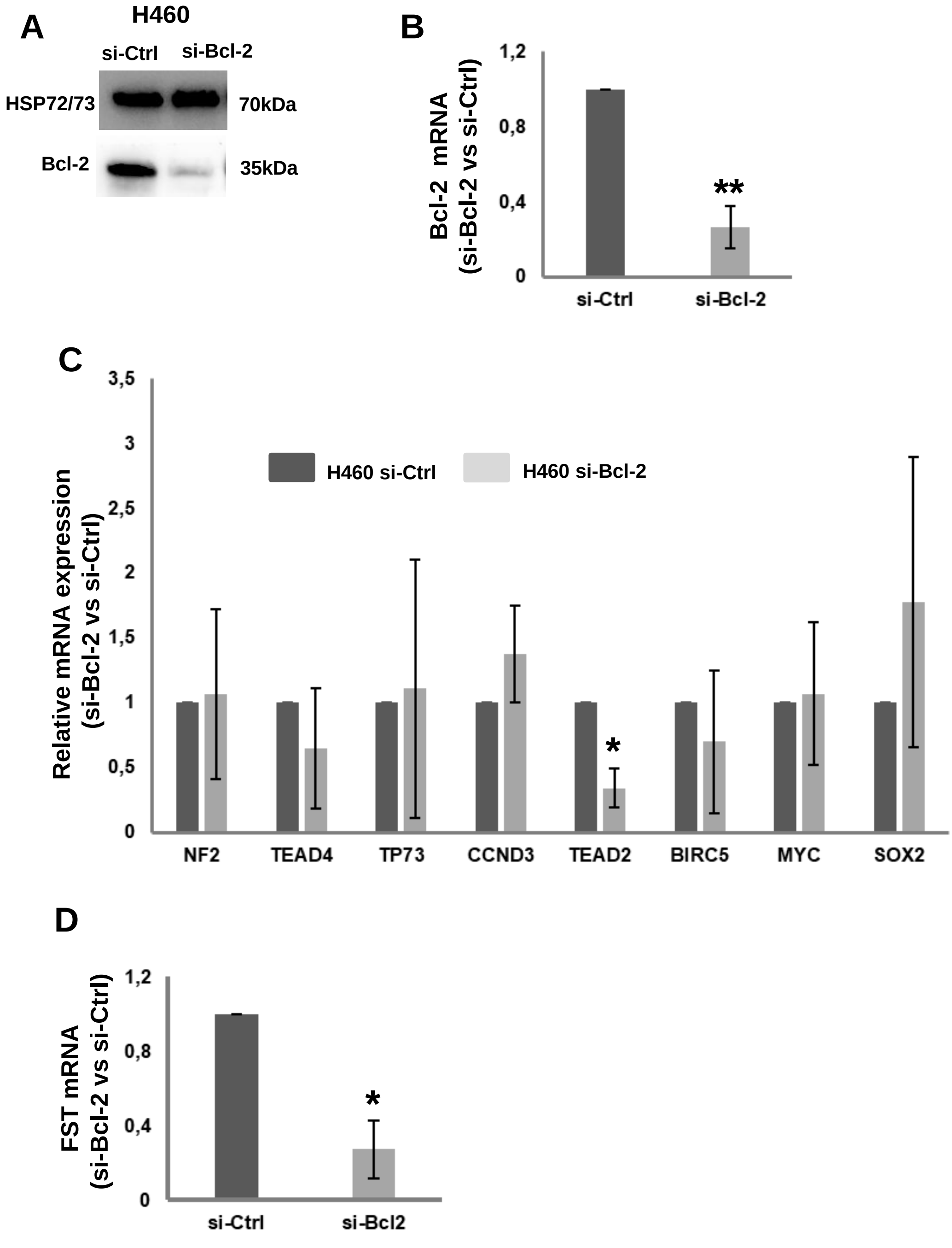

Supplementary Figure S4

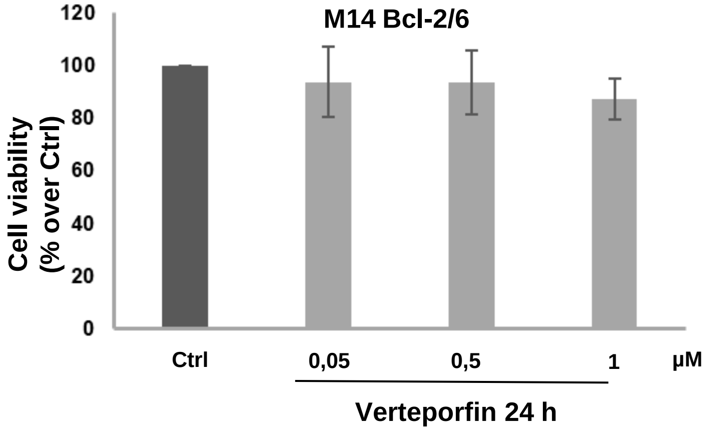

Supplementary Figure S5

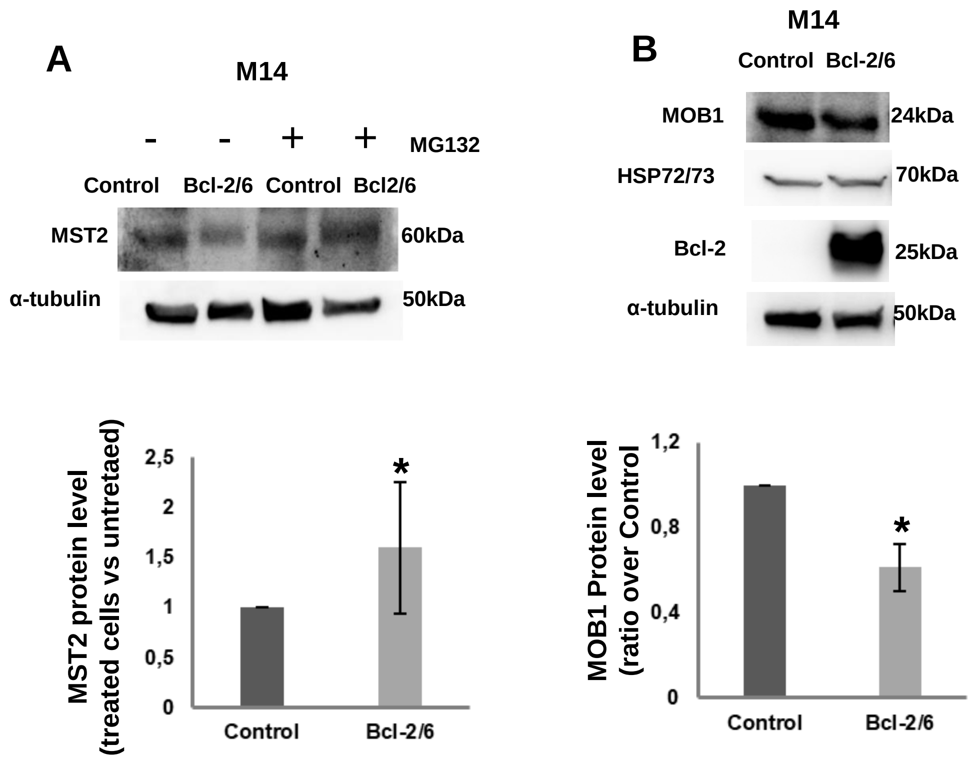

Supplementary Figure S6

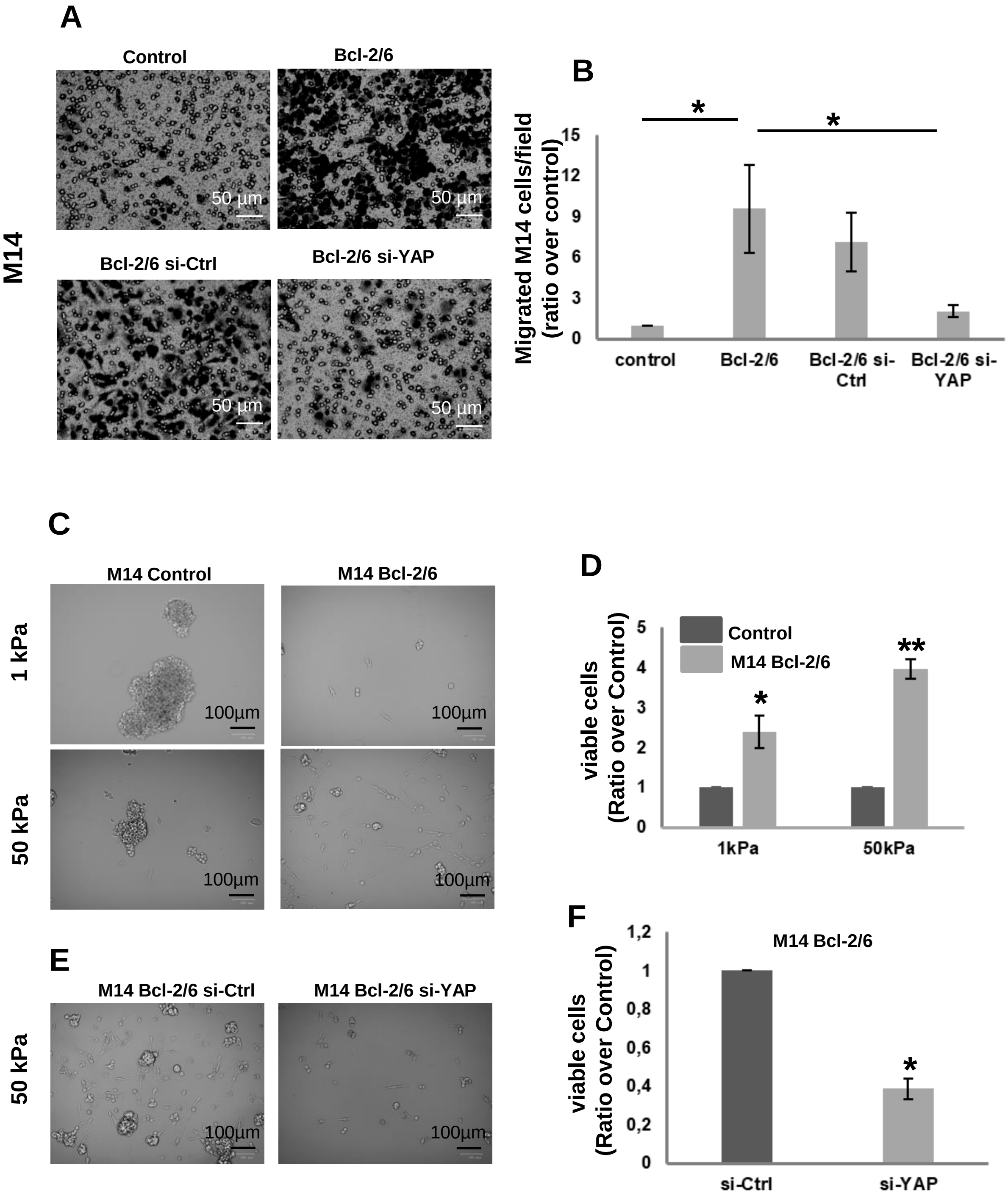

Supplement: Supplementary file 2 — Supplementary Material 2 [file 12964_2024_1647_MOESM2_ESM.pdf]
