# Supplementary material for: Bcl-2 dependent modulation of Hippo pathway in cancer cells
Source: Cell Commun Signal. 2024 May 16;22:277. doi: 10.1186/s12964-024-01647-1 (PMC11097437; doi:10.1186/s12964-024-01647-1)
Supplement: Supplementary file 3 — Supplementary Material 3 [file 12964_2024_1647_MOESM3_ESM.pdf]

Supplementary Figure Legends

Fig.S1 Generation of siRNA-mediated transient Bcl-2 or Bcl-xL knockdown in melanoma cells. Human melanoma A375 cells were transiently transfected with siRNA SMARTpools targeting either Bcl-2 (si-Bcl-2) or Bcl-xL(si-Bcl-xL) or with control siRNA (si-Ctrl). **A** Western blot analysis of Bcl-2 protein expression and **B** qRT-PCR analysis of Bcl-2 mRNA level in A375 si-Bcl-2 or si-Ctrl condition. **C** Western blot analysis of Bcl-xL protein expression and **D** qRT-PCR analysis of Bcl-xL mRNA level in A375 si-Bcl-xL or si-Ctrl conditions. **A**,**C** Representative images of three independent experiments with similar results. HSP72/73 and β-actin were employed to check equal loading and transfer**. B,D** Data represent ratio (mean ± standard deviation) of mRNA expression normalized to β-actin gene in silenced cell condition versus controls from three independent biological repeats. Statistical analysis was performed applying unpaired two-tailed student's t test, $*p < 0.05$, $**p < 0.01$.

Fig.S2 Top enriched GO terms of Bcl-xL-downregulated (**A**) or up-regulated (**B**) genes in A375 cells, as determined by KEGG database.

Fig.S3 Validation of genes modulated by Bcl-2 in H460 human non-small cell lung cancer cells. **A** Western blot analysis of Bcl-2 protein expression and **B** qRT-PCR analysis of Bcl-2 mRNA level in H460 cells transiently transfected with siRNA SMARTpools targeting Bcl-2 (si-Bcl-2) or control siRNA (si-Ctrl). **C** qRT-PCR analysis of NF2, TEAD4, TP73, CCND3, TEAD2, BIRC5, MYC and SOX2. **D** FST mRNA expression in H460 cells si-Bcl-2 respect to si-Ctrl. **A** Representative image of three independent experiments with similar results. HSP72/73 was used to check equal loading and transfer. **B-D** Data represent ratio (mean ± standard deviation) of mRNA expression normalized to β-actin in silenced cell condition versus controls from three independent biological repeats. **A-D** Statistical analysis was performed applying unpaired two-tailed student's t test, $*p < 0.05$, $**p < 0.01$.

Fig.S4. Analysis of cell viability by MTT assay in Bcl-2 overexpressing M14 cells (M14 Bcl-2/6) treated for 24 h with concentrations ranging from 0 to 1 μM of Verteporfin. The results are reported as "viability of treated cells/viability of control cells (Ctrl)" × 100. Data are reported as mean of three experiments (± standard deviation). $*p < 0.05$.

Fig.S5. Bcl-2 regulates the Hippo pathway core proteins. **A** Western blot analysis of MST2 (upper panel) and relative densitometric analysis (lower panel) in control (Control) and Bcl-2-overexpressing (Bcl-2/6) M14 clones in the presence or absence of 10 μM MG132 for 6 h. **B** Western blot analysis of MOB1 protein (upper panel) and relative densitometric analysis in M14 control and Bcl-2/6 clones. The expression level of Bcl-2 protein in the stable clones was also verified. Representative image of three independent experiments with similar results. HSP72/73 and α-tubulin were used to check equal loading and transfer. Statistical analysis was performed applying unpaired two-tailed student's t test, $*p < 0.05$.

Fig.S6. YAP mediates the ability of Bcl-2 to promote cell migration and cell viability in higher stiffness condition. **A** Representative images and **B** relative quantification of *in vitro* cell migration of control (Control) and Bcl-2 overexpressing (Bcl-2/6) M14 clones and M14 Bcl-2/6 clone transiently transfected with siRNA SMARTpools targeting YAP (si-YAP) or with control siRNA (si-Ctrl). The values are reported as ratio (mean ± standard deviation) of number of migrated cells/field versus control. The quantification was performed by counting the number of migrated cells in at least 5 fields for each condition. **C** Representative images and **D** relative quantification of viability of Control and M14 Bcl-2/6 clones seeded on 1kPa and 50kPa stiffness. The values are reported as ratio (mean ± standard deviation) of viable Bcl-2/6 cells over control. **E** Representative images and **F** relative quantification of viability of Bcl-2/6 cells seeded on 50kPa stiffness and transiently transfected with si-YAP or with si-Ctrl. The values are reported as ratio (mean ± standard deviation) of viable cells over control. **C-F** Cell viability was assessed by Trypan blue staining and using an automatic cell counter. **A-F** Experiments have been performed in three biological replicates. Statistical analysis was performed applying unpaired two-tailed student's t test, $*p < 0.05$, $**p < 0.01$, ns= not significant.
