# Supplementary material for: Bcl-2 dependent modulation of Hippo pathway in cancer cells
Source: Cell Commun Signal. 2024 May 16;22:277. doi: 10.1186/s12964-024-01647-1 (PMC11097437; doi:10.1186/s12964-024-01647-1)

Fig.2B

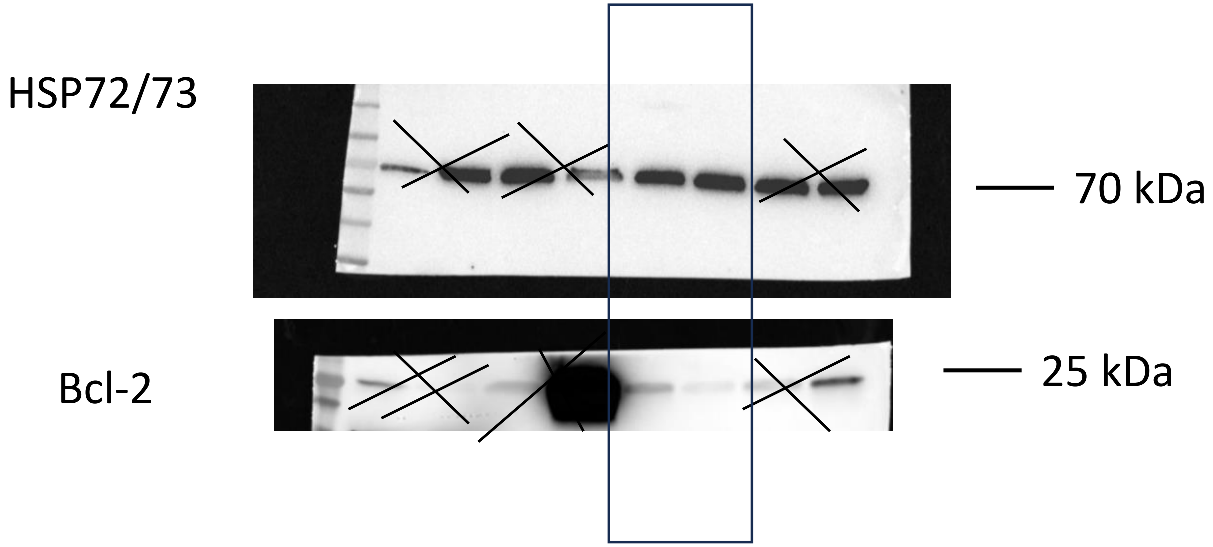

Fig.3D

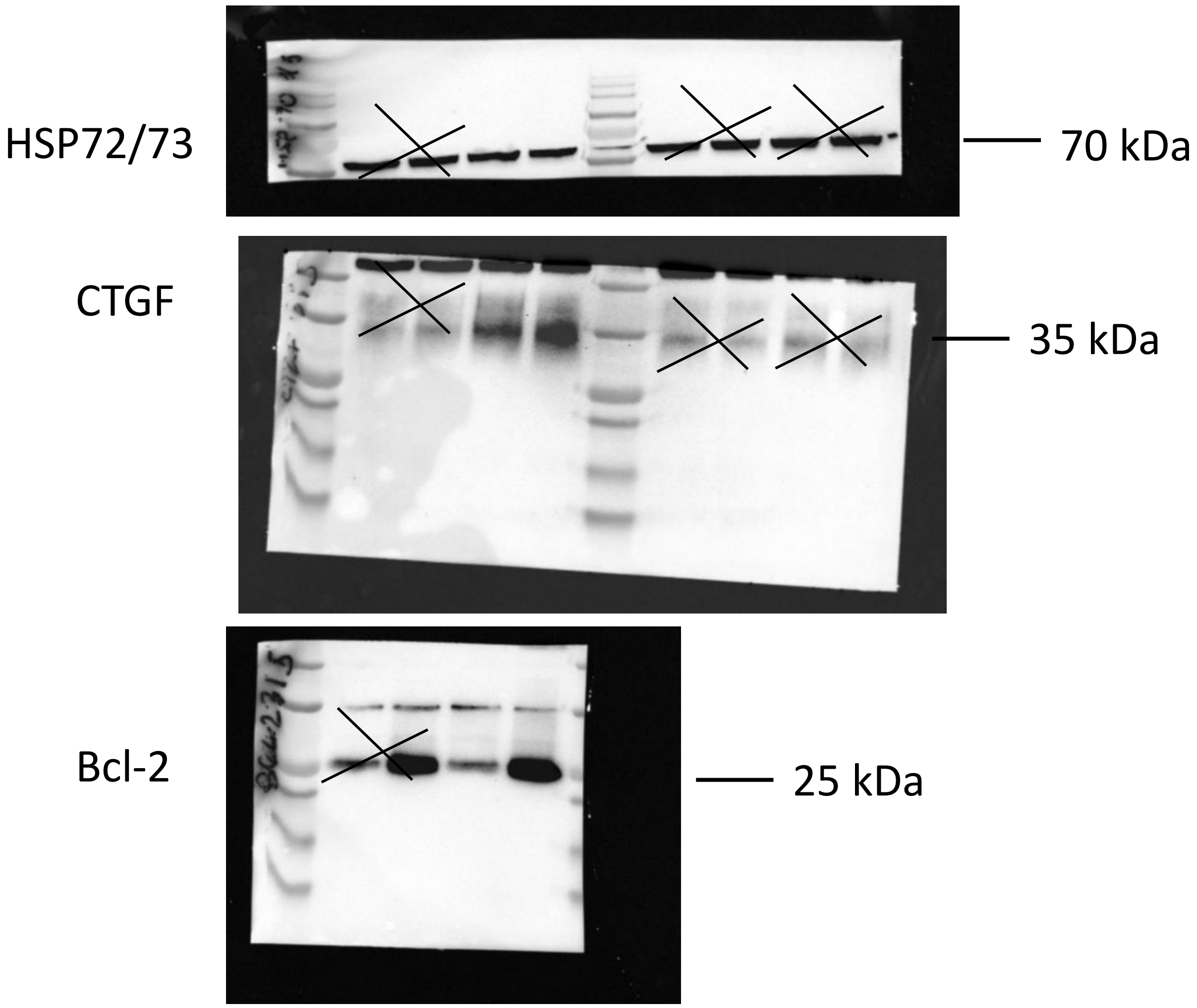
HSP72/73
70 kDa
CTGF
35 kDa
Bcl-2
25 kDa

Fig.3F

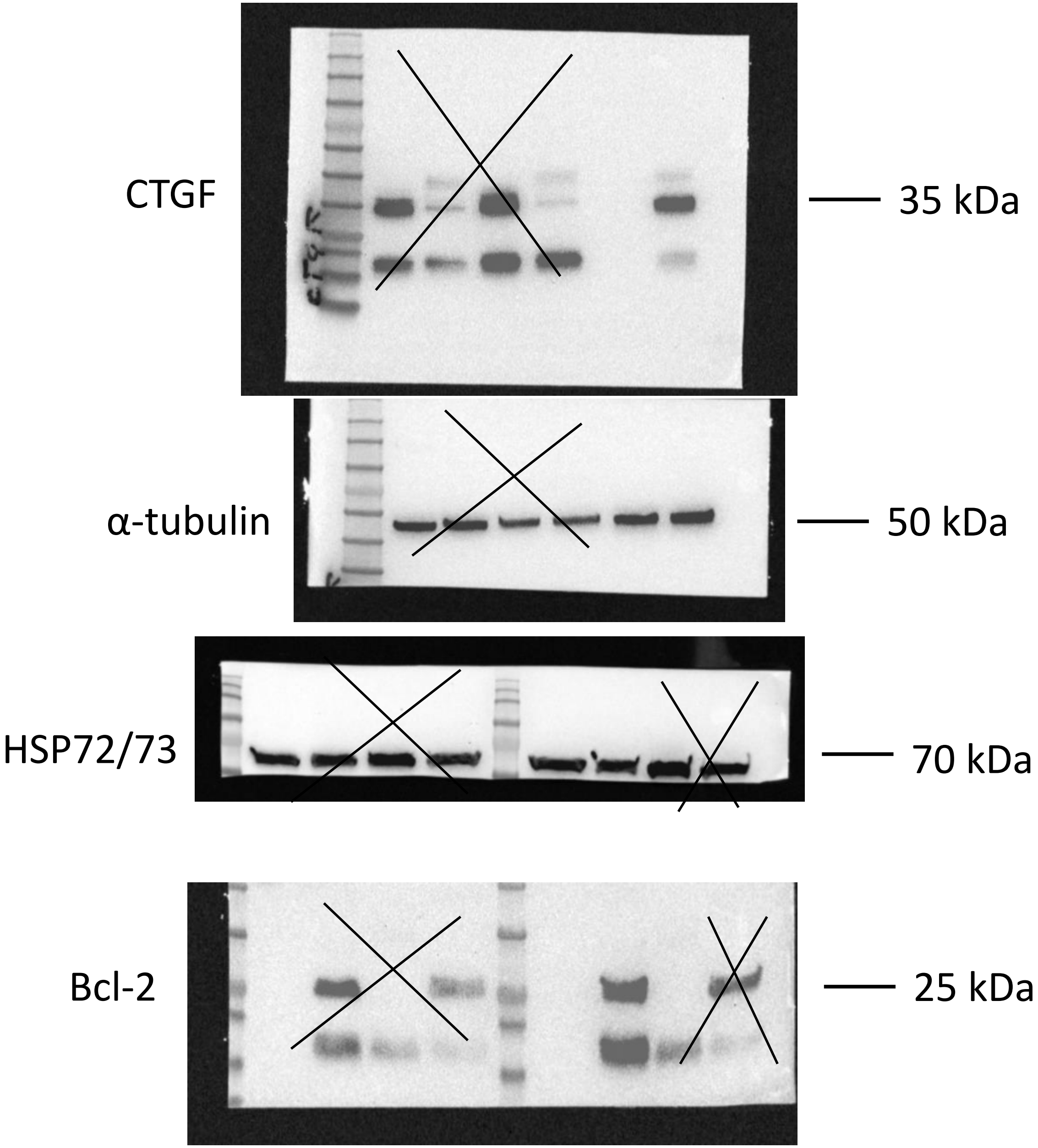
CTGF
35 kDa
α-tubulin
50 kDa
HSP72/73
70 kDa
Bcl-2
25 kDa

Fig.3H

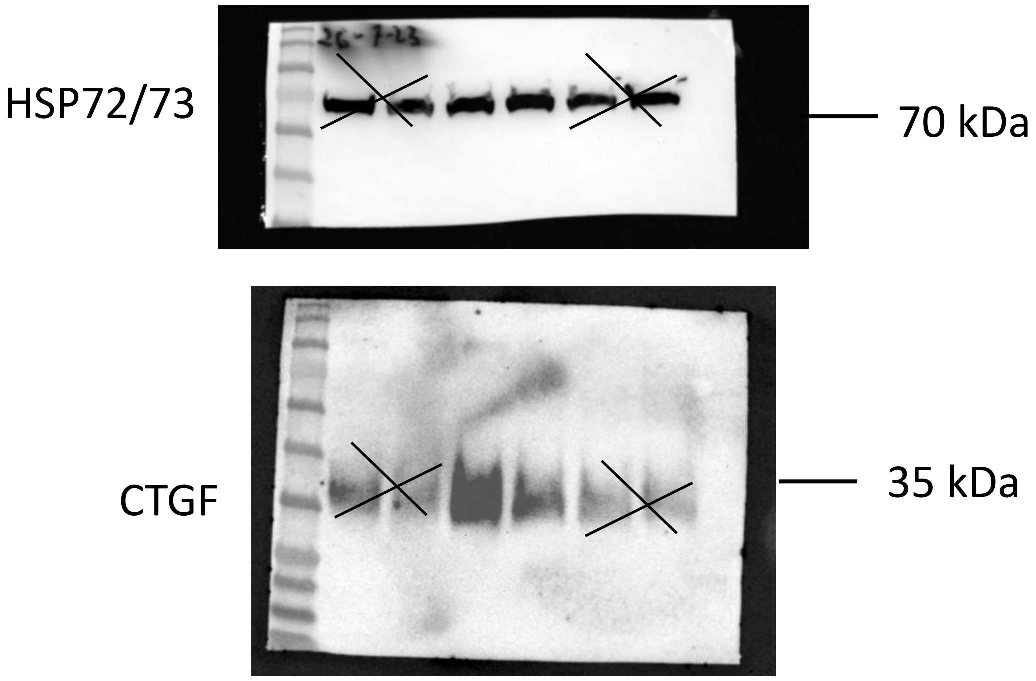

Fig.4A

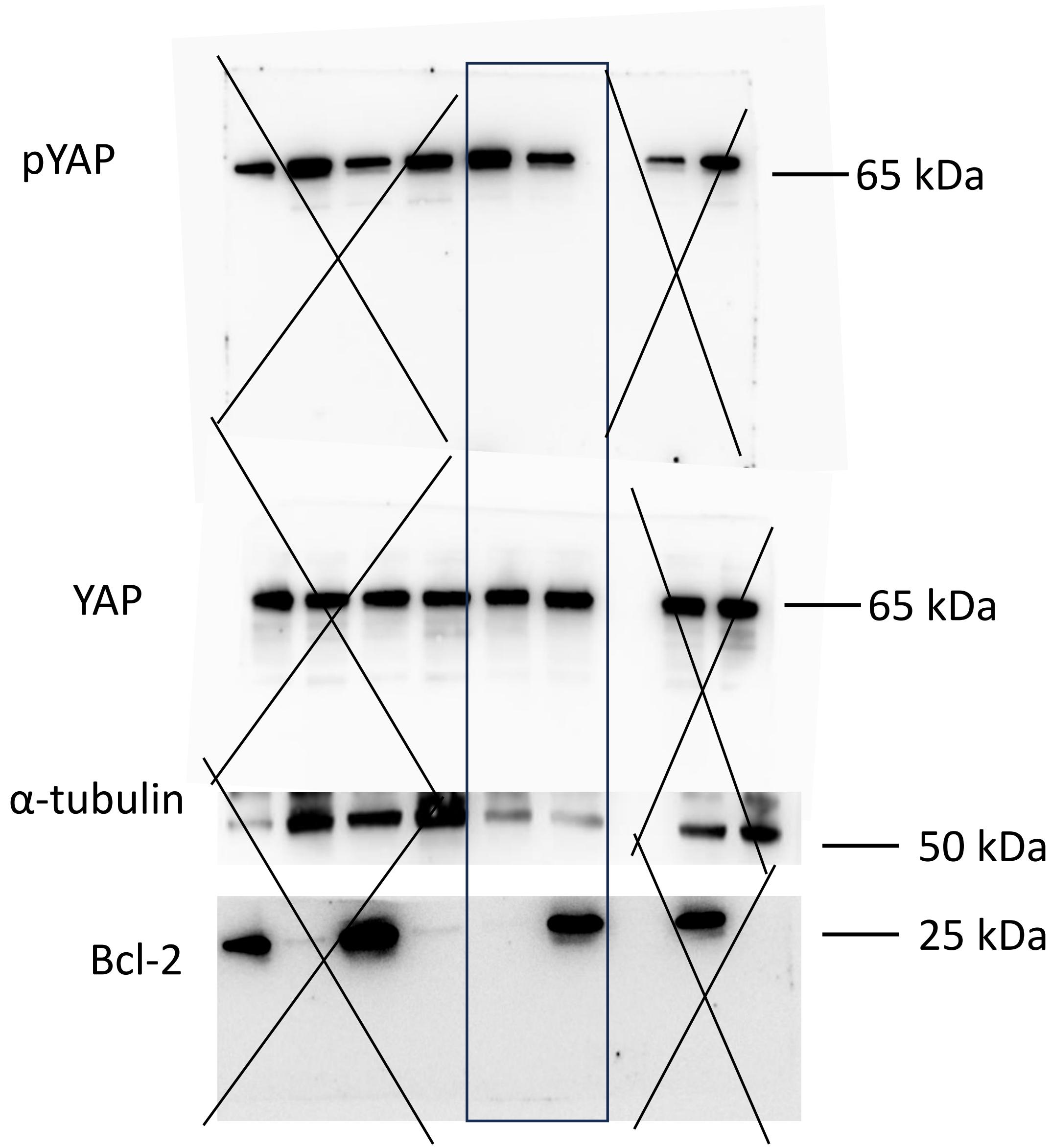

Fig.4B

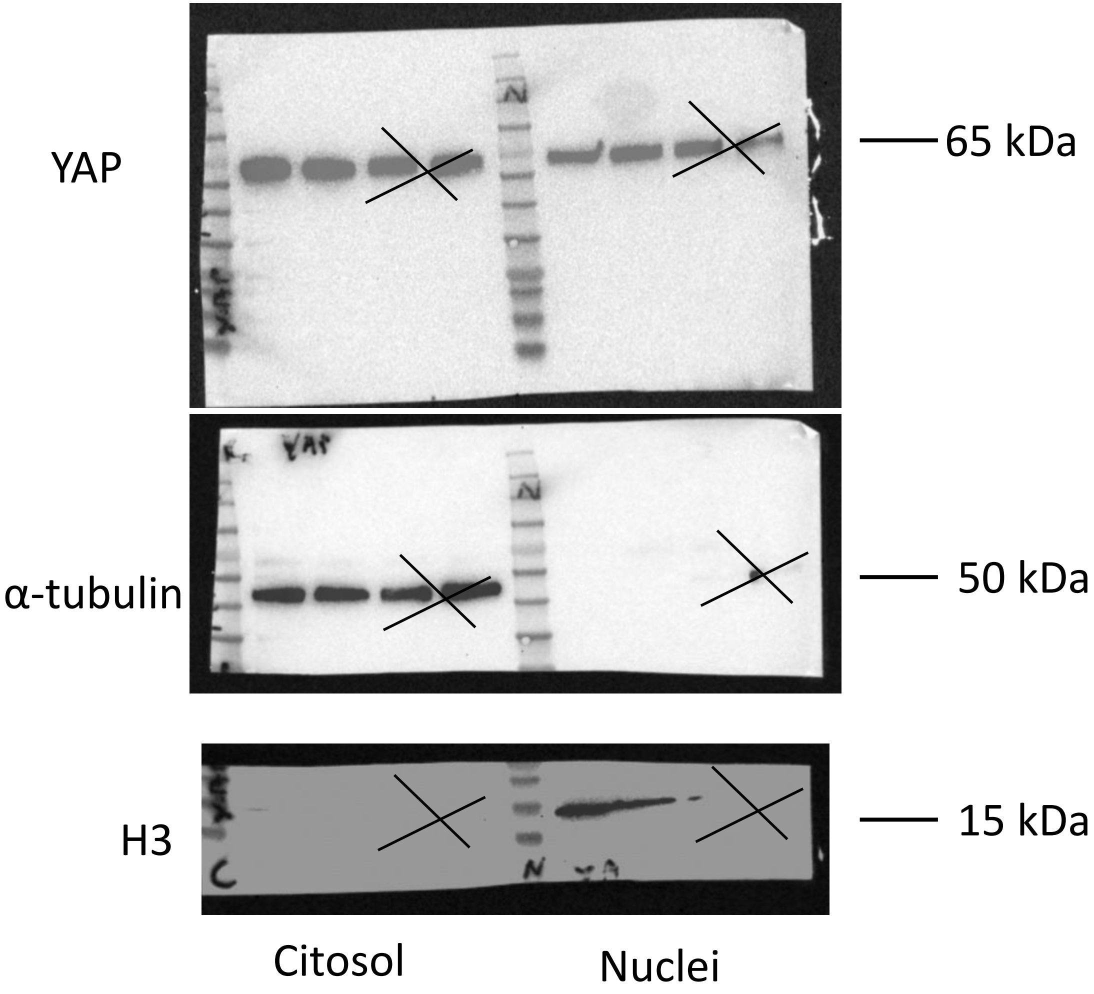
YAP
65 kDa
α-tubulin
50 kDa
H3
15 kDa
Citosol
Nuclei

Fig.4C

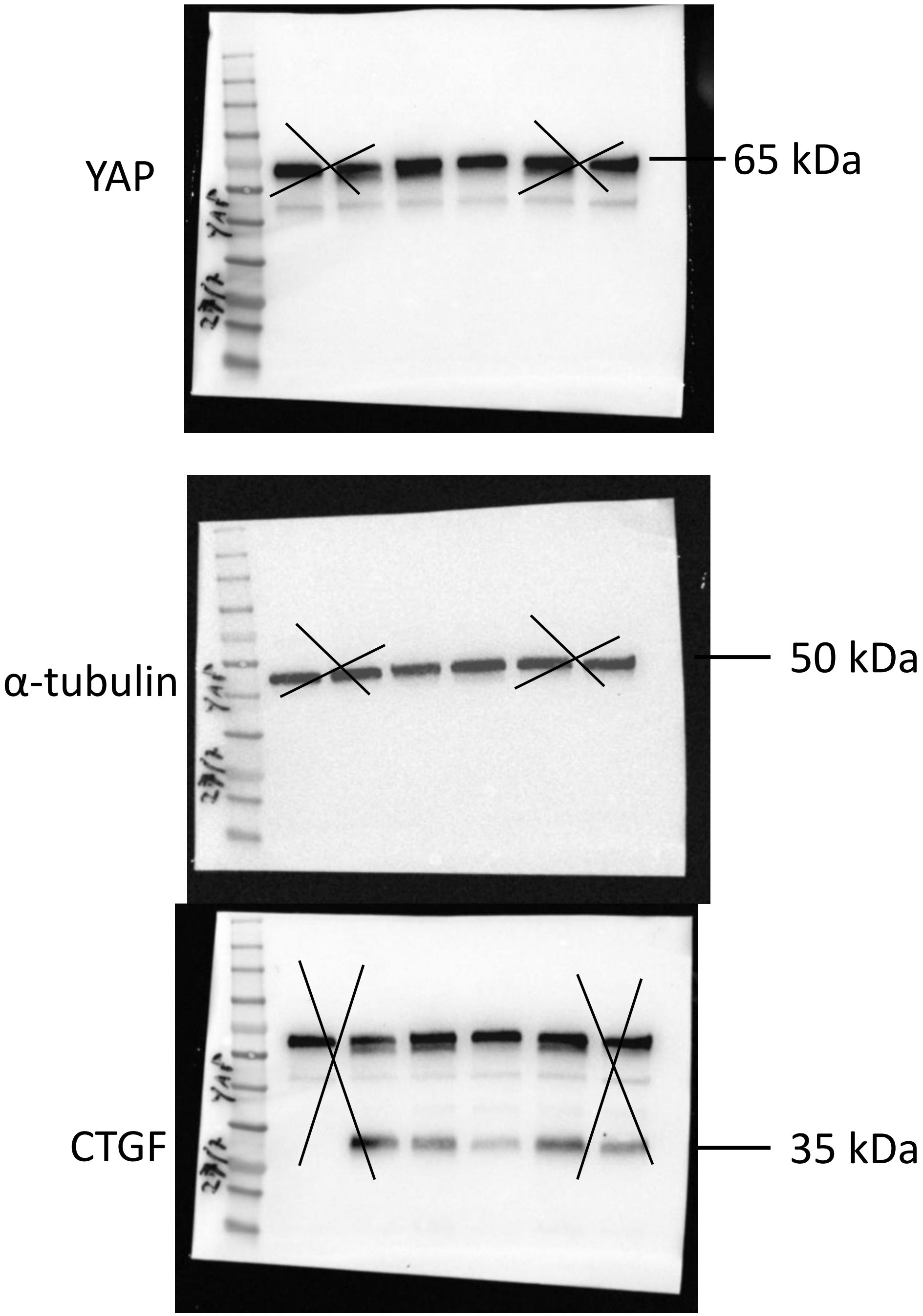
YAP
65 kDa
α-tubulin
50 kDa
CTGF
35 kDa

Fig.4E

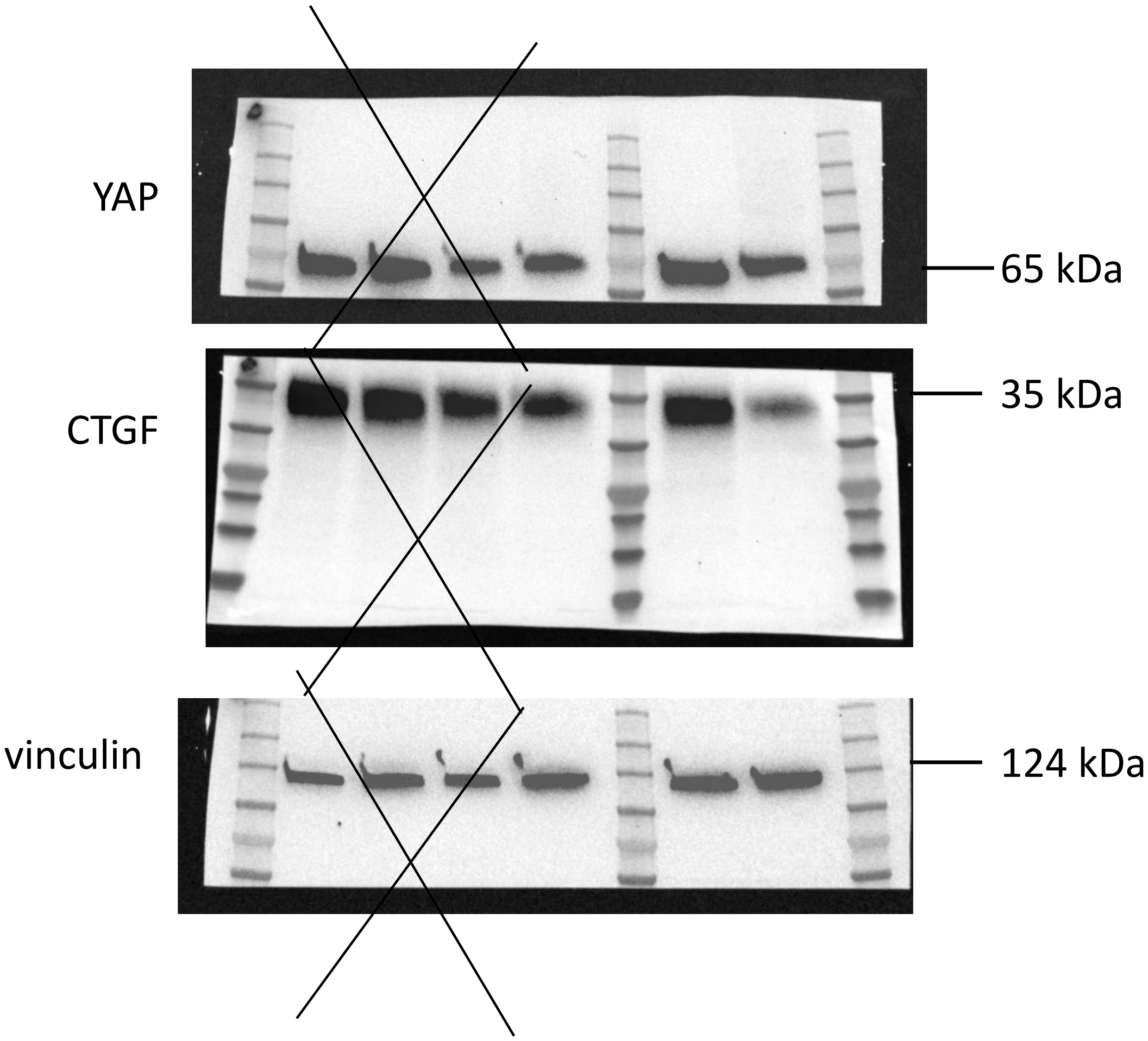

Fig.5A

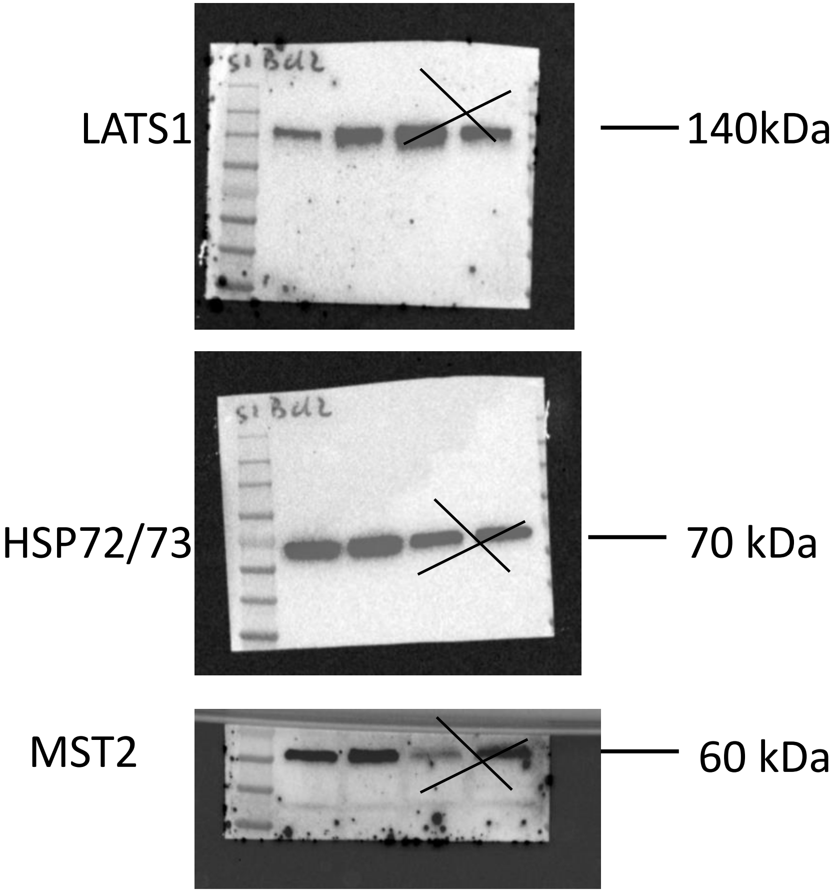

Fig.5B

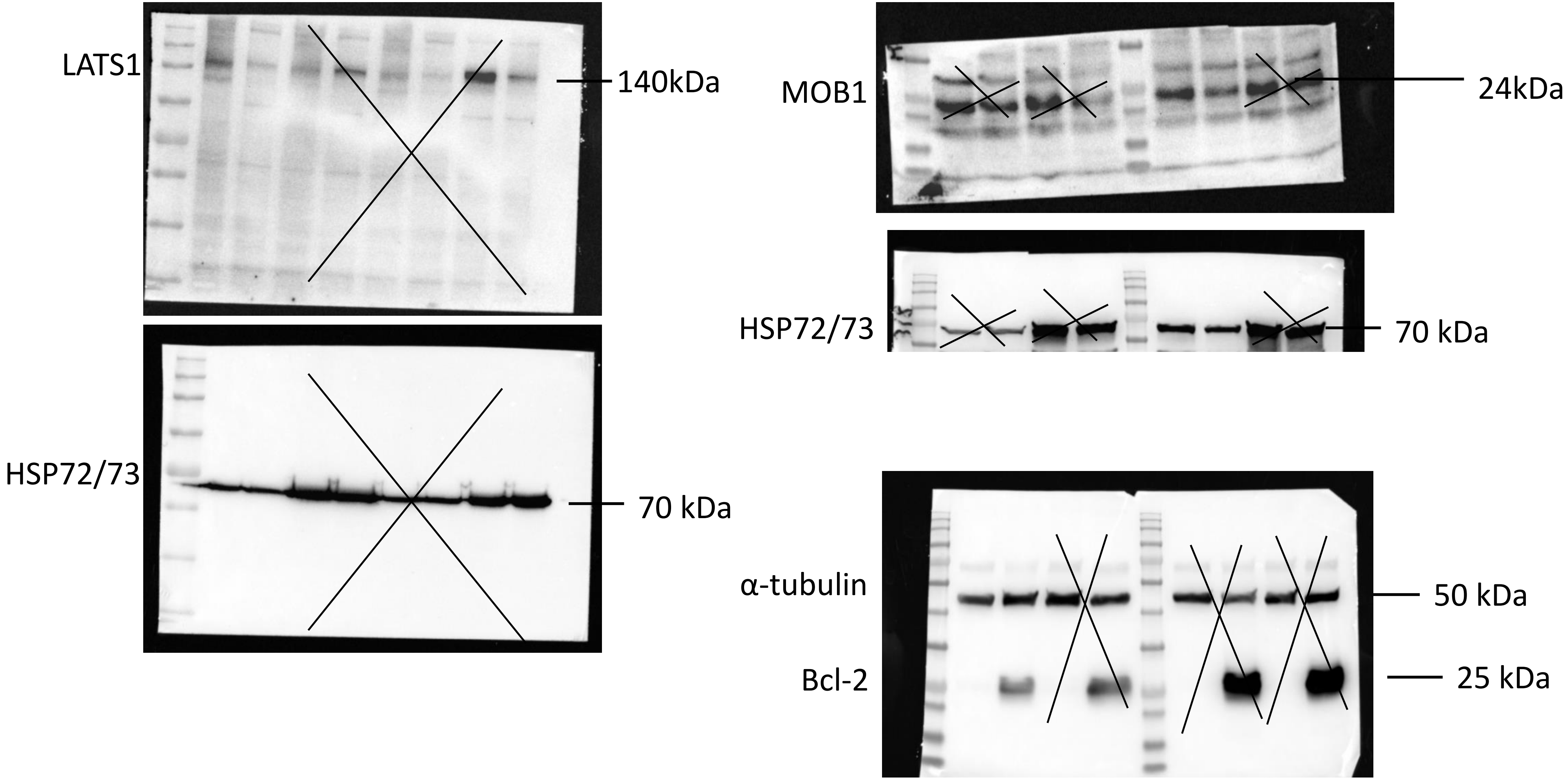
LATS1
140kDa
HSP72/73
70 kDa
MOB1
24kDa
HSP72/73
70 kDa
α-tubulin
50 kDa
Bcl-2
25 kDa

Fig.5C

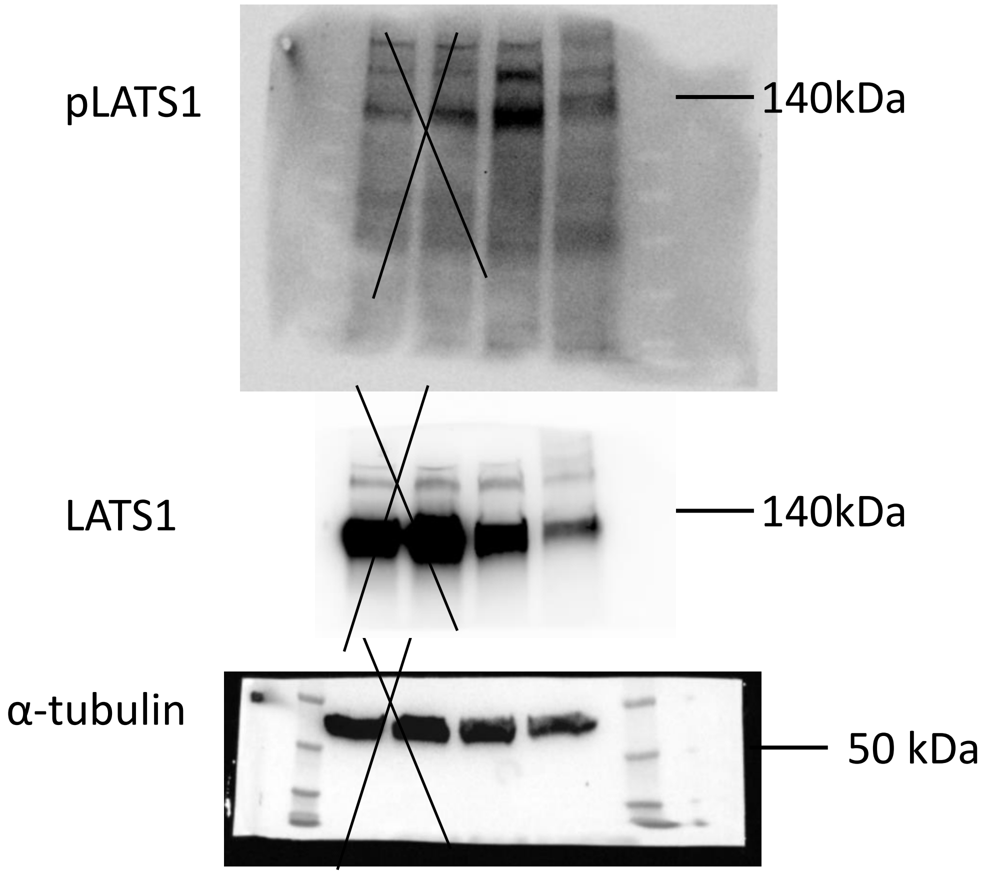

Fig.5D

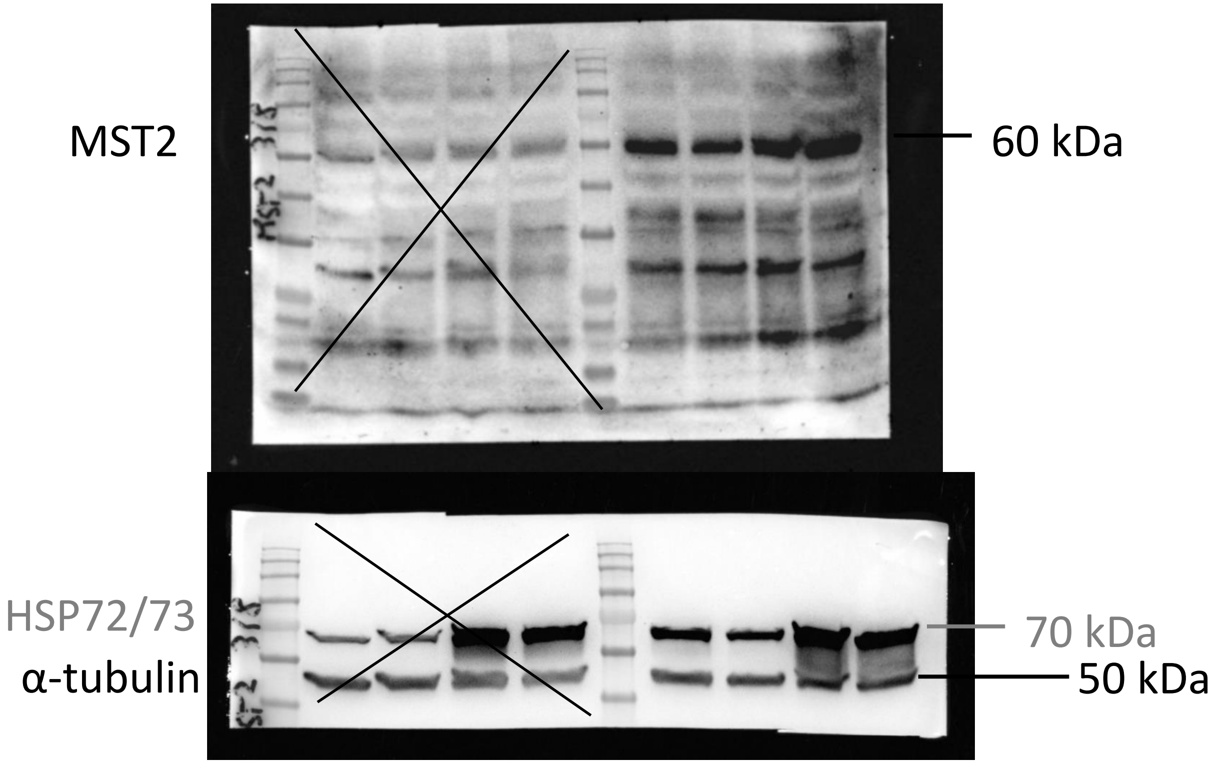

Fig.5E

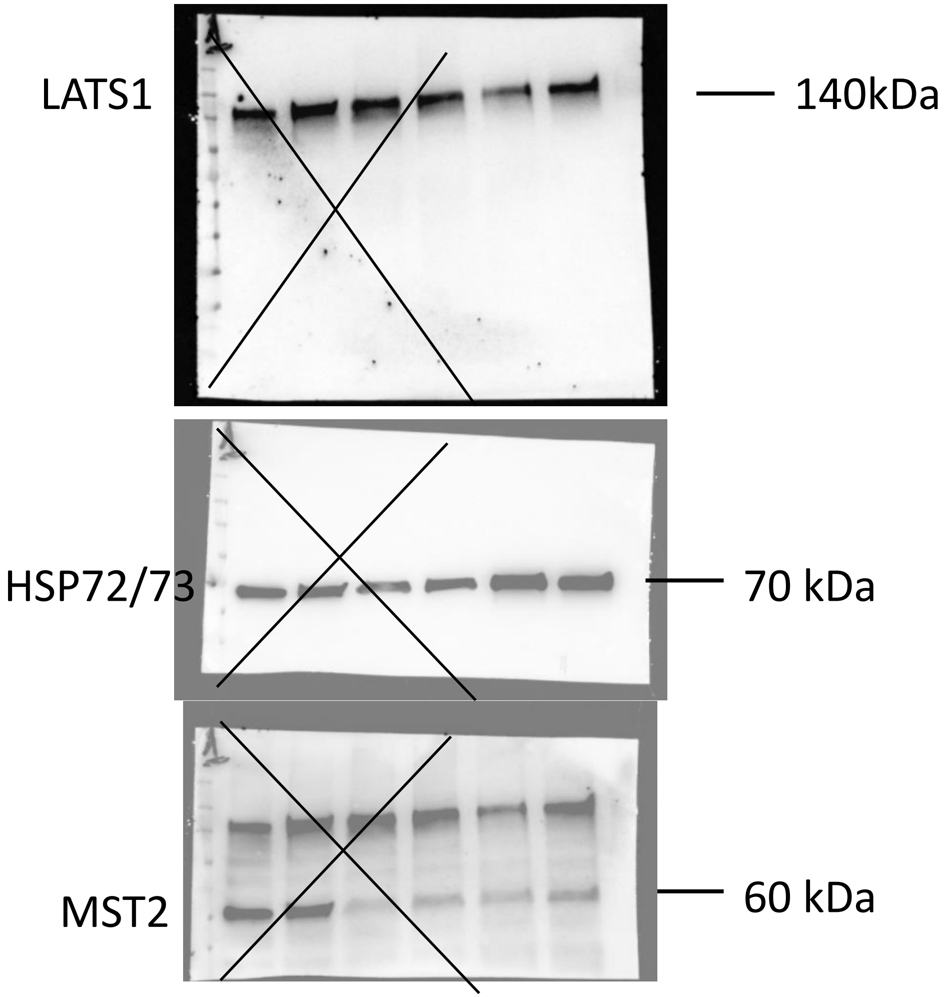

Fig.5F

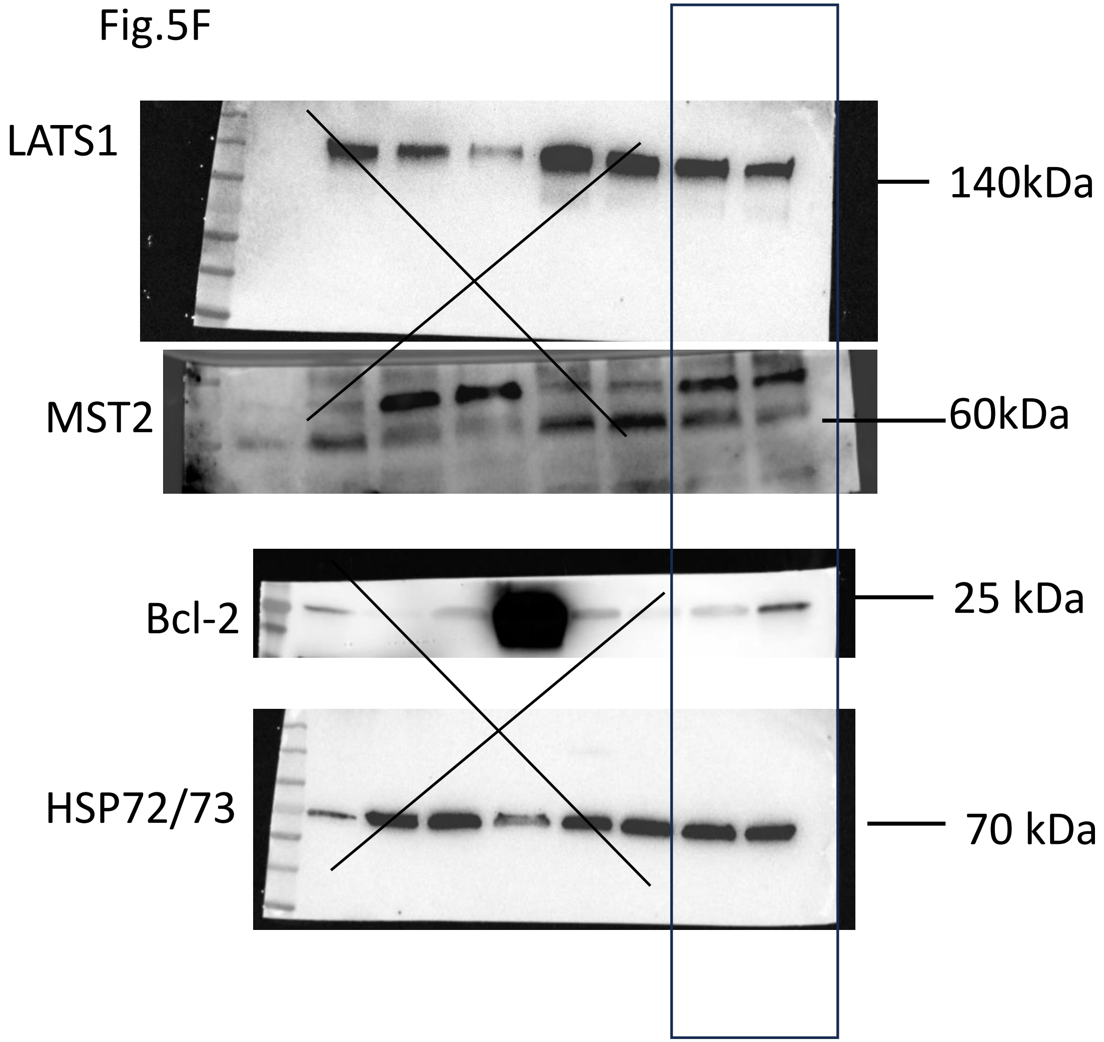

Fig.8C

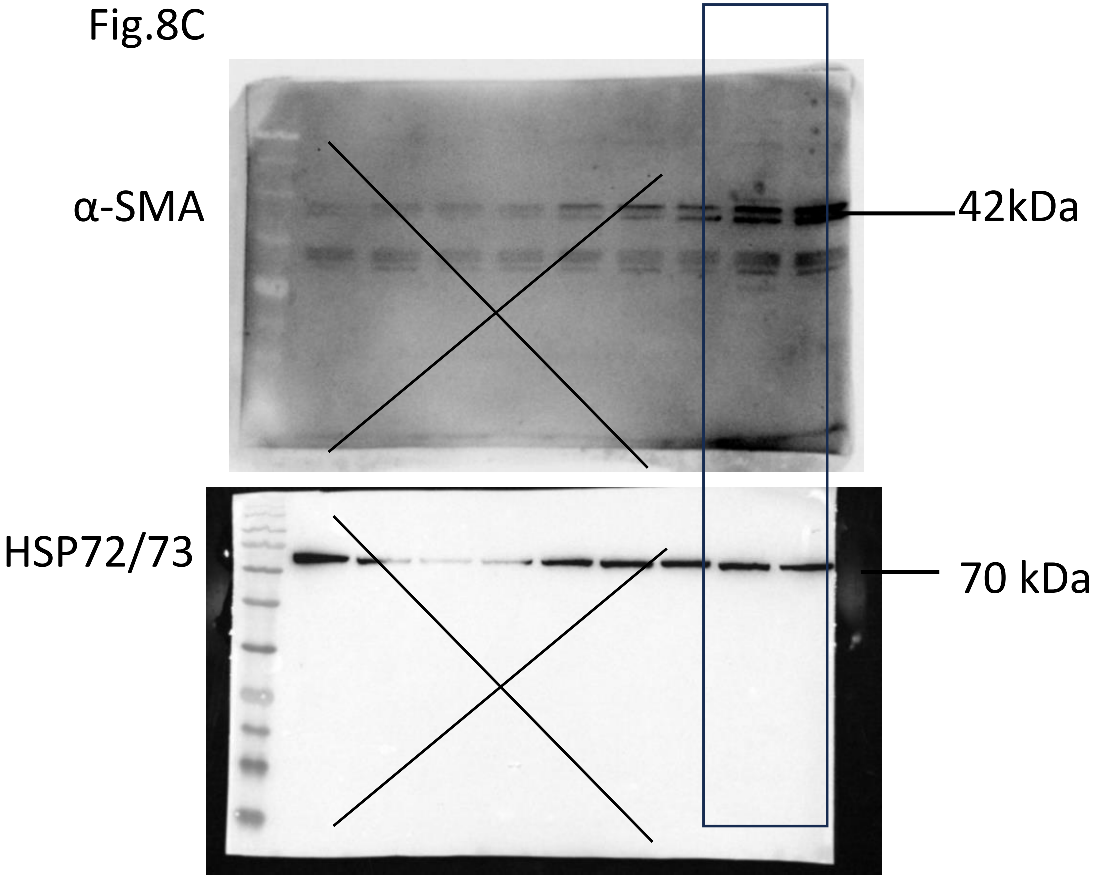
α-SMA
42kDa
HSP72/73
70 kDa

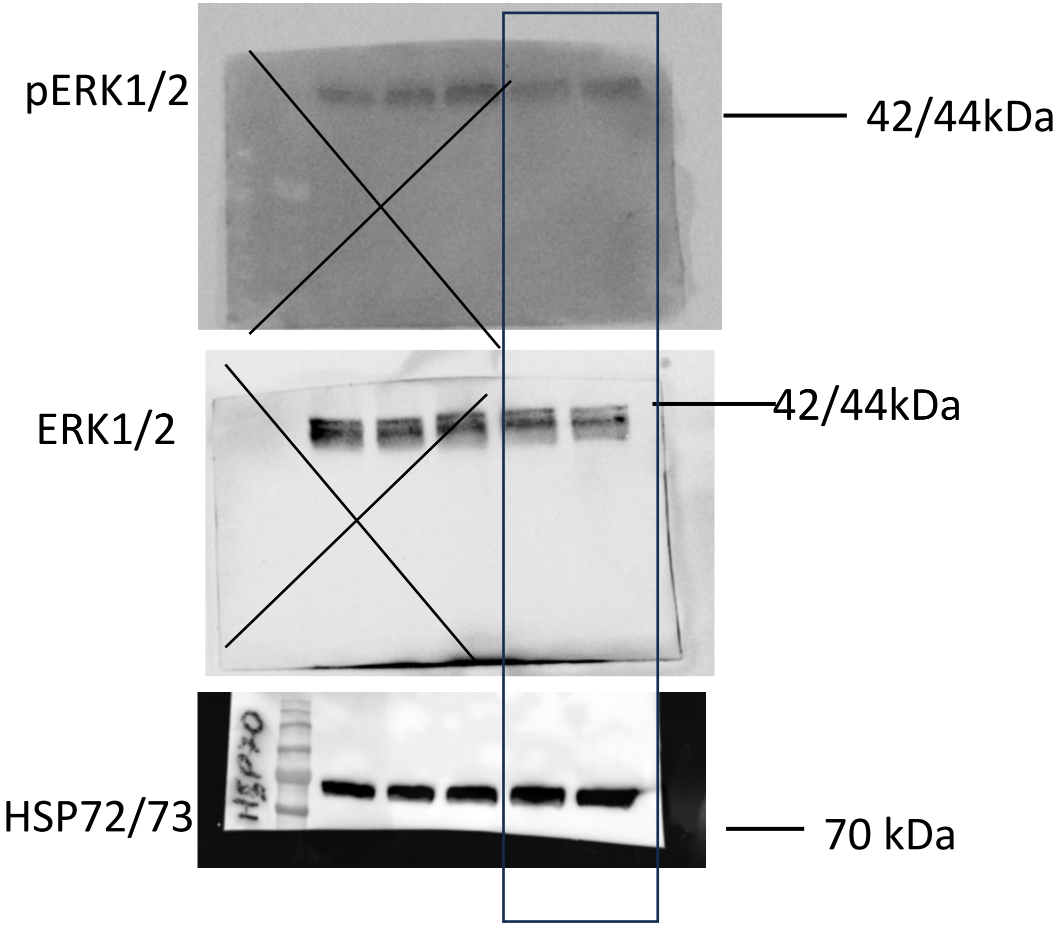
pERK1/2
42/44kDa
ERK1/2
42/44kDa
HSP72/73
70 kDa

Fig.9B

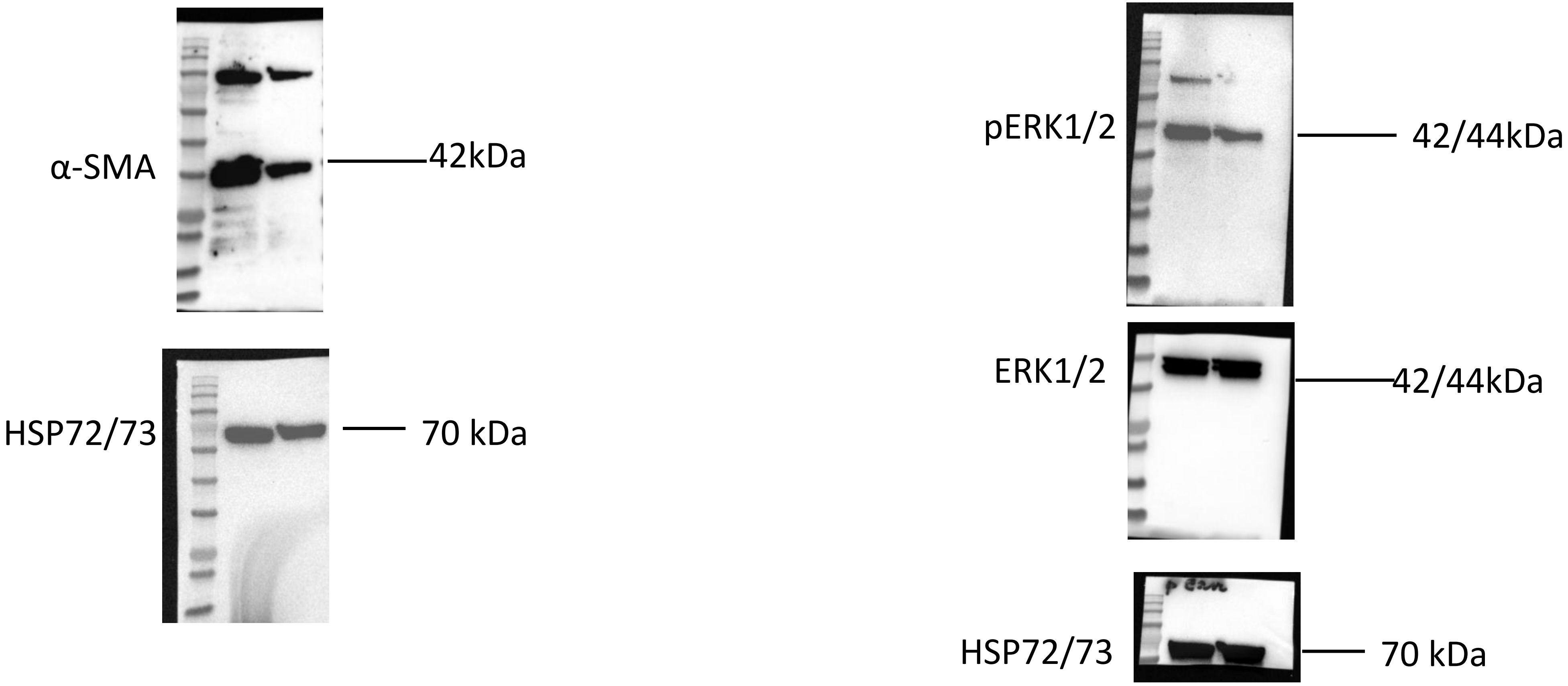
α-SMA
42kDa
HSP72/73
70 kDa
pERK1/2
42/44kDa
ERK1/2
42/44kDa
HSP72/73
70 kDa

Supplementary Figure 1A

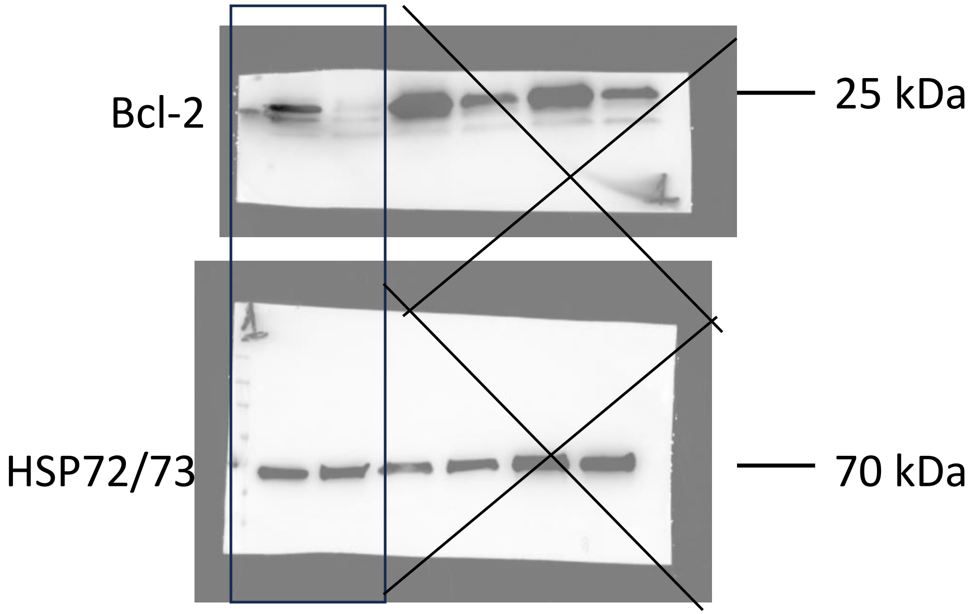

Supplementary Figure 1C

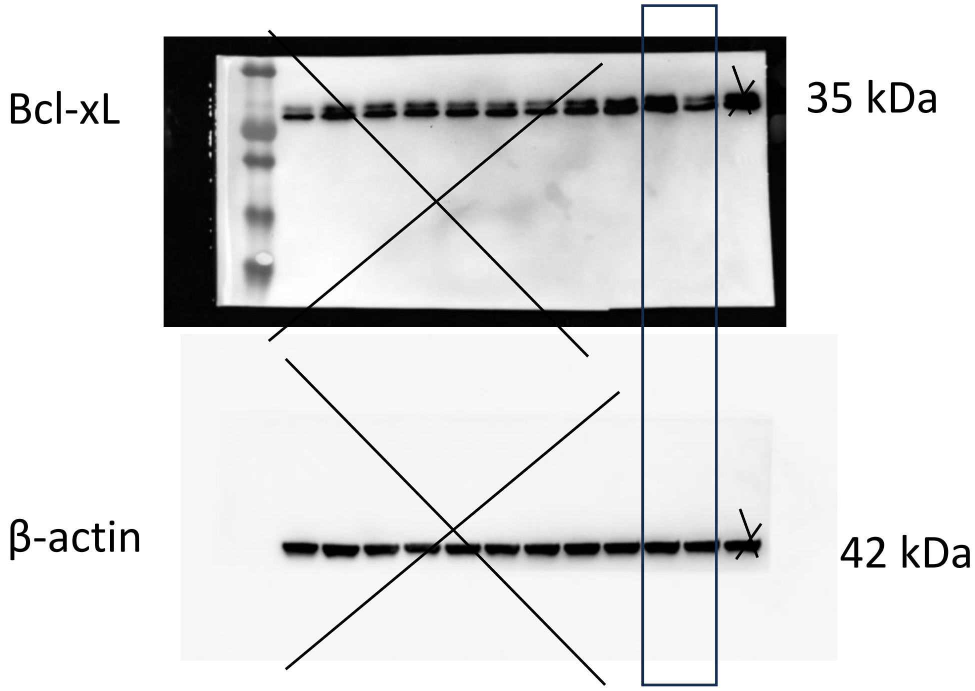

Supplementary Figure 3A

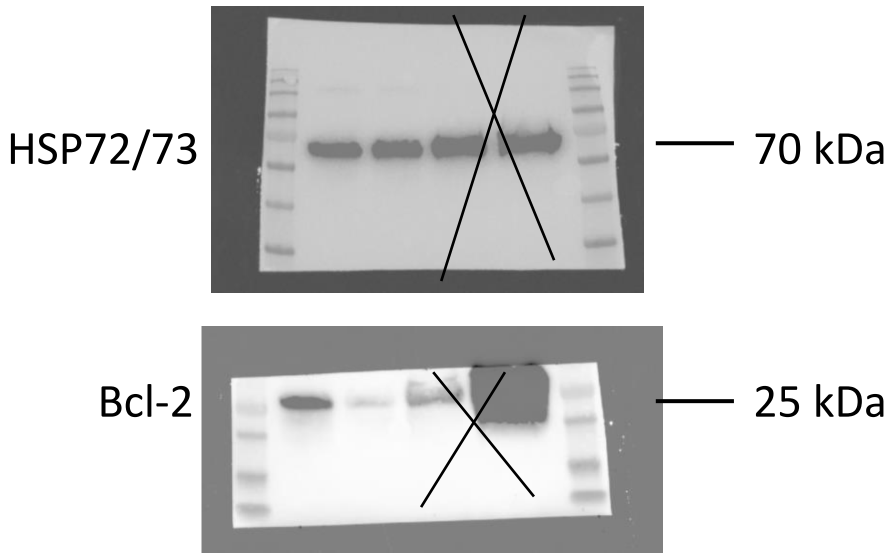

Supplementary Figure 4B

Supplementary Figure 4A

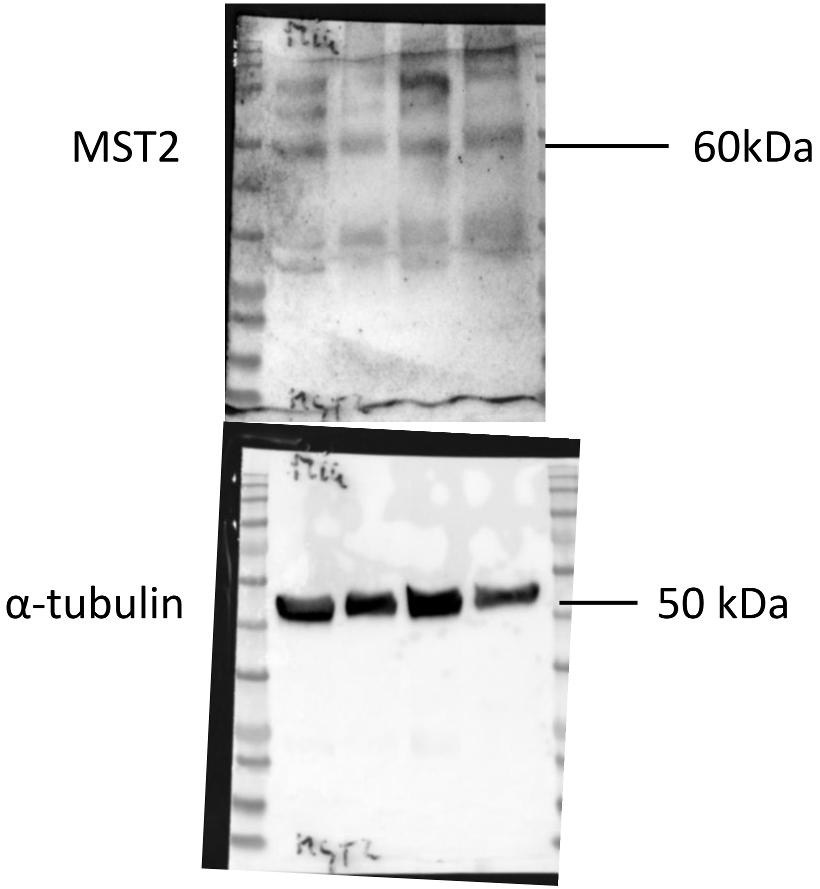

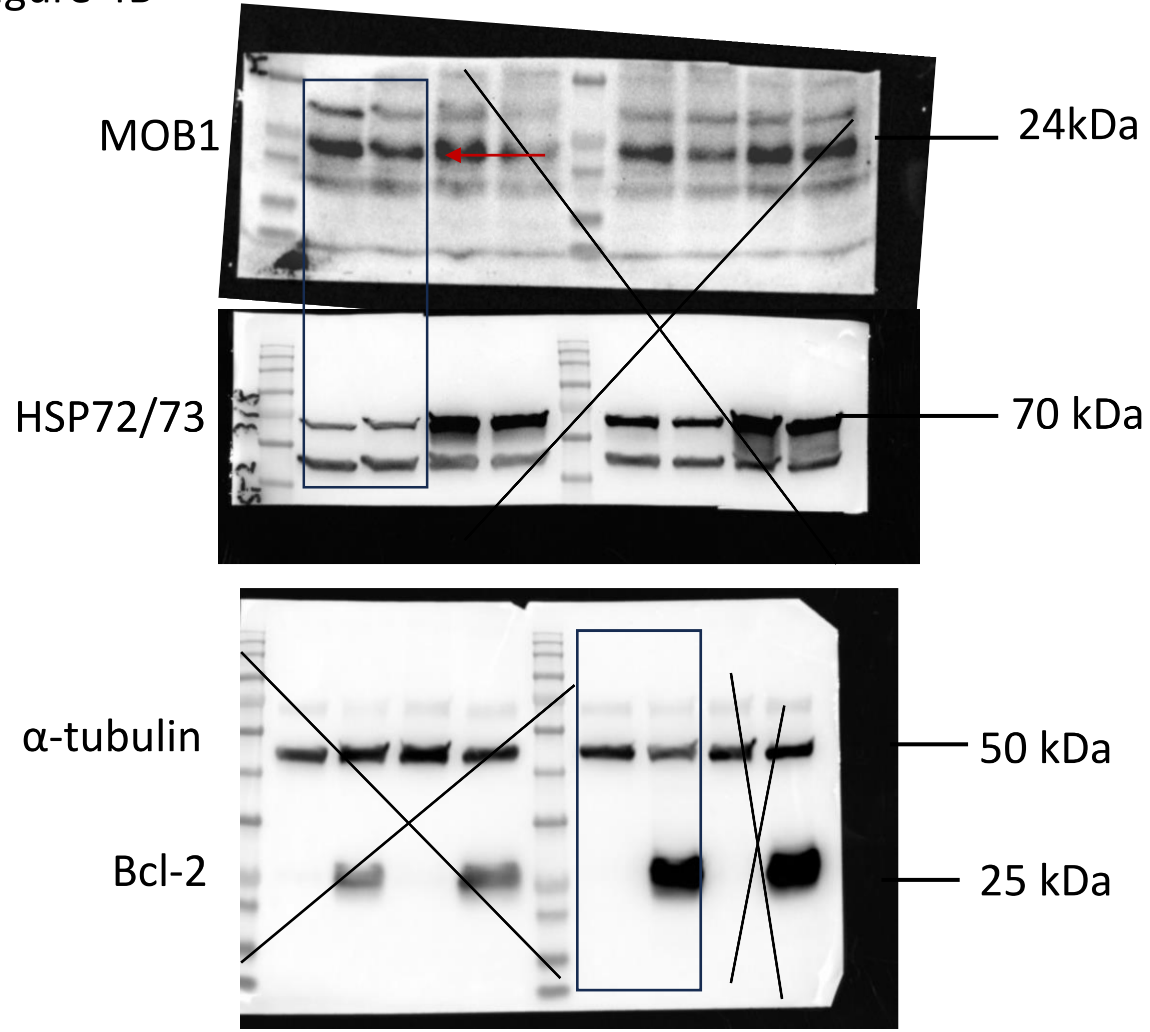

Supplement: Supplementary file 6 — Supplementary Material 6 [file 12964_2024_1647_MOESM6_ESM.pdf]
